# GCN2 kinase activation mediates pulmonary vascular remodeling and pulmonary arterial hypertension

**DOI:** 10.1172/jci.insight.177926

**Published:** 2024-09-24

**Authors:** Maggie M. Zhu, Jingbo Dai, Zhiyu Dai, Yi Peng, You-Yang Zhao

**Affiliations:** 1 Program for Lung and Vascular Biology, Section for Injury Repair and Regeneration Research, Stanley Manne Children’s Research Institute, Ann & Robert H. Lurie Children’s Hospital of Chicago, Chicago, Illinois, USA.; 2 Department of Pediatrics, Division of Critical Care, Northwestern University Feinberg School of Medicine, Chicago, Illinois, USA.; 3 Genetic Medicine and Nanotechnology Development Center (GeneMeNDer), Stanley Manne Children’s Research Institute, Ann & Robert H. Lurie Children’s Hospital of Chicago, Chicago, Illinois, USA.; 4 Departments of Pharmacology and Medicine and; 5 Feinberg Cardiovascular and Renal Research Institute, Northwestern University Feinberg School of Medicine, Chicago, Illinois, USA.

**Keywords:** Vascular biology, Cardiovascular disease, Endothelial cells, Protein kinases

## Abstract

Pulmonary arterial hypertension (PAH) is characterized by progressive increase of pulmonary vascular resistance and remodeling that result in right heart failure. Recessive mutations of *EIF2AK4* gene (encoding general control nonderepressible 2 kinase, GCN2) are linked to heritable pulmonary veno-occlusive disease (PVOD) in patients but rarely in patients with PAH. The role of GCN2 kinase activation in the pathogenesis of PAH remains unclear. Here, we show that GCN2 was hyperphosphorylated and activated in pulmonary vascular endothelial cells (ECs) of hypoxic mice, monocrotaline-treated rats, and patients with idiopathic PAH. Unexpectedly, loss of GCN2 kinase activity in *Eif2ak4^–/–^* mice with genetic disruption of the kinase domain induced neither PVOD nor pulmonary hypertension (PH) but inhibited hypoxia-induced PH. RNA-sequencing analysis suggested endothelin-1 (Edn1) as a downstream target of GCN2. GCN2 mediated hypoxia-induced Edn1 expression in human lung ECs via HIF-2α. Restored Edn1 expression in ECs of *Eif2ak4^–/–^* mice partially reversed the reduced phenotype of hypoxia-induced PH. Furthermore, GCN2 kinase inhibitor A-92 treatment attenuated PAH in monocrotaline-treated rats. These studies demonstrate that GCN2 kinase activation mediates pulmonary vascular remodeling and PAH at least partially through Edn1. Thus, targeting GCN2 kinase activation is a promising therapeutic strategy for treatment of PAH in patients without *EIF2AK4* loss-of-function mutations.

## Abstract 2

GCN2 kinase activated in pulmonary endothelial cells of hypoxic mice, monocrotaline rats, and PAH patients mediates pulmonary vascular remodeling and PAH via HIF-2a-regulated endothelin-1 expression

## Introduction

Pulmonary hypertension (PH) is defined by a resting mean pulmonary arterial pressure greater than 20 mmHg (1). Although there are many causes of PH, the development of PH is almost always associated with exacerbating symptoms and increased mortality, regardless of the underlying disease (2). Based on pathophysiological mechanisms, clinical presentation, hemodynamic characteristics, and therapeutic management, PH is clinically classified into 5 groups (1). Pulmonary arterial hypertension (PAH) (group 1) is characterized by a progressive increase in pulmonary vascular remodeling and pulmonary vascular resistance, resulting in right ventricle hypertrophy and, ultimately, right heart failure (3–5). Over the past few decades, progress has been made for the treatment of PAH and substantially improved the prognosis of patients with PAH (6–9). However, currently approved PAH medications are mainly focused on reducing pulmonary vascular constriction and only provide partial symptomatic relief (10). Due to the poor understanding of the molecular mechanisms of pulmonary vascular cell dysfunction leading to the progressive vascular remodeling, there is a lack of treatment directly targeting pulmonary vascular remodeling, and thus the mortality rate of PAH remains unacceptably high, which is still 50% at 5 years after diagnosis (11).

General control nonderepressible 2 kinase (GCN2), a serine-threonine kinase found in all eukaryotic organisms, is primarily known as a sensor of metabolic stress such as limited amino acids, glucose, or purine (12–16). In response to amino acid starvation, GCN2 is activated by the binding of uncharged tRNAs accumulated in amino acid–starved cells to the histidyl-tRNA synthetase–related (HisRS-related) domain of GCN2 (17–19) inducing conformational changes (20, 21) and autophosphorylation (22) and subsequently phosphorylates the subunit of eukaryotic translation initiation factor 2α subunit (EIF2α) to suppress general protein synthesis but selectively activate stress protein synthesis for adaptation to amino acid starvation (14). Phosphorylation in the activation loop of GCN2, especially at yeast GCN2 amino acid Thr887 equivalent to mouse Thr898 and human Thr899, is required for GCN2 activation (22), and it locks the kinase domain in an open active form, which can bind substrate, such as EIF2α, in the absence of tRNA (21–25). In addition to sensing nutritional deprivation, recent studies have shown that GCN2 can also be activated by several other stresses, including UV irradiation (26), oxidative stress (27), and hypoxia (28). Apart from the role in regulating translation initiation, GCN2 has also been implicated in affecting G1 arrest and apoptosis (28), tumor growth (29), and inflammation (30).

Recessive mutations in the *EIF2AK4* gene (encoding GCN2) and resultant reduction of GCN2 protein expression are linked to heritable pulmonary veno-occlusive disease (PVOD), a rare subgroup of severe PAH, which is characterized by intimal proliferation and fibrosis of septal veins and preseptal venules (31). The PVOD subgroup has a worse prognosis than the classical PAH group, and currently there are no effective treatments in addition to lung transplantation (32). Biallelic *EIF2AK4* mutations are found in 25% of histologically confirmed sporadic cases of PVOD (31). Although *EIF2AK4* mutation is rarely identified in idiopathic PAH (IPAH) (9 of 864 patients with PAH) (33), *EIF2AK4* mutation is also identified in some patients with heritable PAH but not in family members without PAH (34). Most of these mutations are stop codons or insertions/deletions that disrupt gene function, indicating that loss of GCN2 function induces PVOD or PAH without PVOD in patients.

Here we sought to determine whether *Eif2ak4^–/–^* (KO) mice with disruption of the kinase domain develop spontaneous PVOD and the role of GCN2 signaling in pulmonary vascular remodeling and PAH development. To our surprise, the KO mice did not develop spontaneous PVOD and PH. Thus, we challenged the mice with chronic hypoxia. Unexpectedly, loss of Gcn2 in the KO mice attenuated PH. Endothelium-targeted nanoparticle delivery (35) of endothelin-1 (Edn1) plasmid DNA to restore Edn1 expression in endothelial cells (ECs) of *Gcn2*-deficient mice partially reversed the reduced phenotype of hypoxia-induced PH. GCN2 kinase inhibitor A-92 treatment also inhibited monocrotaline-induced PH in rats. In patients with IPAH, we observed prominent GCN2 phosphorylation at Thr899, i.e., GCN2 activation in pulmonary vascular ECs. Our studies, which we believe to be novel, demonstrate a detrimental role of GCN2 kinase activation in promoting pulmonary vascular remodeling and PAH development. Thus, targeting GCN2 kinase signaling may represent a promising therapeutic approach for treatment of patients with PAH without *EIF2AK4* recessive mutations.

## Results

### Loss of Gcn2 kinase domain induces neither spontaneous PVOD nor PH in mice.

**Figure 1:**
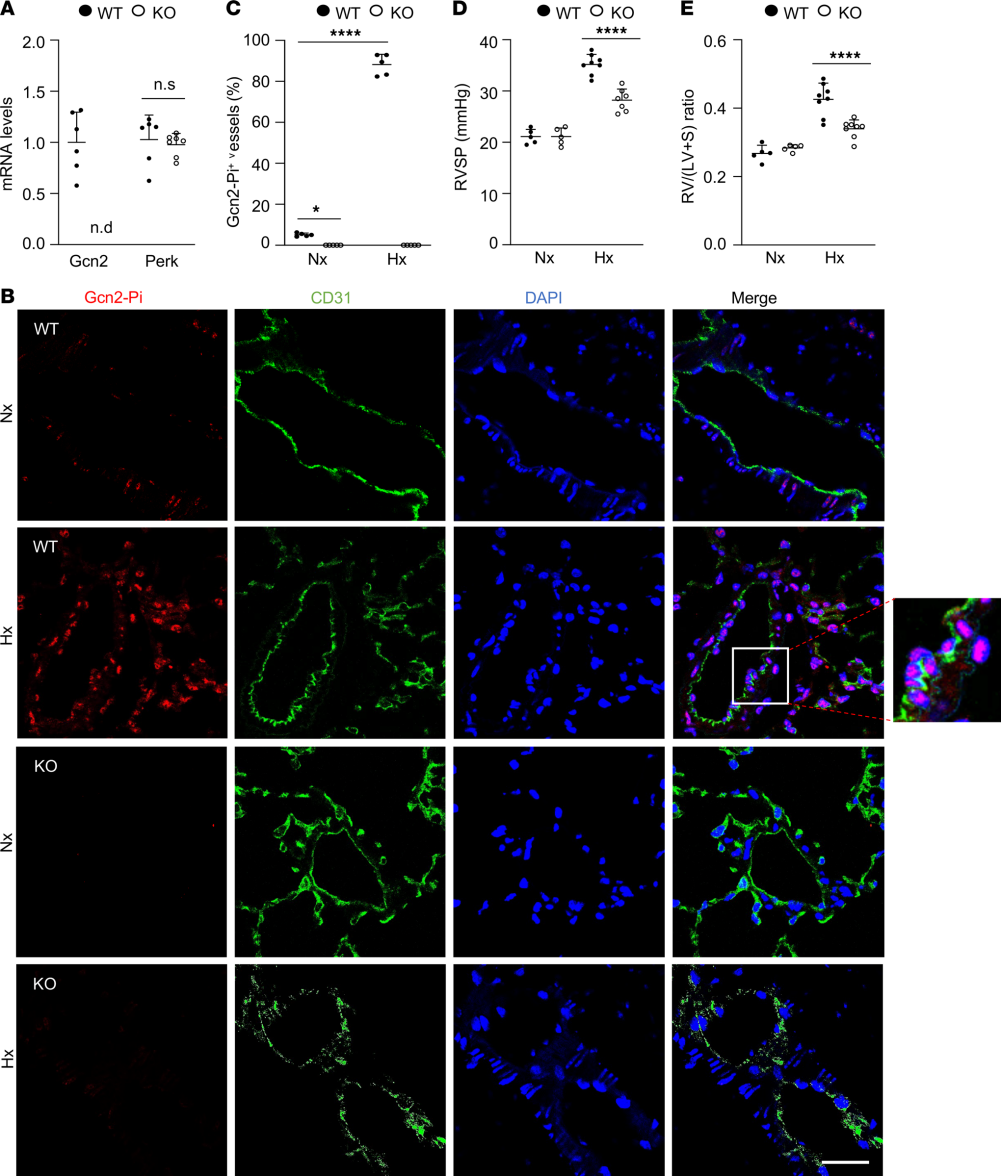
GCN2 deficiency attenuates hypoxia-induced PH in mice. (**A**) Quantitative RT-PCR analysis verifying *Gcn2*-specific disruption in *Eif2ak4^–/–^* (KO) mouse lungs. WT *n* = 6, KO *n* = 7. n.d., not detected; RT, reverse transcription. (**B**) Representative micrographs of anti–phosphorylated (phospho-) GCN2 immunostaining of mouse lungs demonstrating prominent Thr898 phospho-GCN2 (GCN2-Pi) in pulmonary vascular ECs in hypoxic WT but not KO mice. Lung tissue cryosections from normoxic and hypoxic (3 weeks) WT mice and hypoxic KO mice were immunostained with anti-Thr phospho-GCN2 antibody (red). ECs were immunostained with anti-CD31 (green), and nuclei were counterstained with DAPI (blue). Scale bar, 50 μm. (**C**) Quantification of GCN2-Pi–positive vessels. Data are expressed as percentage of positive vessels in each mouse lung. *N* = 5/group. (**D**) RVSP measurement showing increased RVSP in hypoxic WT mice compared with normoxic WT mice, which was reduced in hypoxic KO mice. (**E**) RV hypertrophy evident by increased RV/(LV+S) ratio seen in hypoxic WT mice was reduced in hypoxic KO mice. *N* = 5–8/group. Nx, normoxia; Hx, hypoxia. Data are shown as means + SD. *, *P* < 0.05; ****, *P* < 0.0001. Unpaired 2-tailed *t* test (**A**); 2-way ANOVA with Tukey’s multiple comparisons test (**C**–**E**).

To determine if the KO mice develop spontaneous PVOD or PH, we carried out hemodynamic measurements and histological assessment of pulmonary veno occlusion. As shown in Figure 1A, *Gcn2* mRNA expression was abolished in KO lungs, but the expression of *Perk*, another stress response kinase (28, 36), was not affected, demonstrating gene-specific knockout. At ages of 3.5, 12, and 23 months, right ventricle systolic pressure (RVSP) of KO mice was 23.7 ± 1.3, 23.5 ± 1.3, 24.2 ± 0.8, respectively, compared with 22.6 ± 1.9 in 3.5-month-old WT mice (Supplemental Figure 1A; supplemental material available online with this article; https://doi.org/10.1172/jci.insight.177926DS1). The RV/left ventricle+septum (RV/LV+S) weight ratio, an indicator of RV hypertrophy, of KO mice was also similar to WT mice (Supplemental Figure 1B). These data demonstrate that KO mice do not develop spontaneous PH. Furthermore, H&E staining and Russell-Movat pentachrome staining of these mouse lung sections revealed no evidence of vascular occlusion or luminal narrowing (Supplemental Figure 2), illustrating that KO mice do not have the histopathological features of PVOD.

### Loss of Gcn2 kinase domain protects mice from hypoxia-induced PH.

Since the KO mice do not spontaneously develop PVOD or PH, we challenged the mice with chronic hypoxia to assess their responses to PH development. Immunofluorescence staining and Western blotting revealed prominent Gcn2 phosphorylation at Thr898, indicative of activation (22) in pulmonary vascular ECs of hypoxic WT mice, which was completely absent in KO lungs (Figure 1, B and C, and Supplemental Figure 3). Following 3 weeks of hypoxia (10% O_2_), WT mice developed PH evident by a marked increase of RVSP (35.2 ± 1.9 mmHg), whereas KO mice exhibited a substantially lower RVSP (28.2 ± 2.2 mmHg) (Figure 1D). Hypoxic KO mice also had a decreased RV/LV+S ratio, i.e., RV hypertrophy, compared with hypoxic WT mice (Figure 1E). These data demonstrate that loss of Gcn2 kinase function attenuates hypoxia-induced PH in mice.

**Figure 2:**
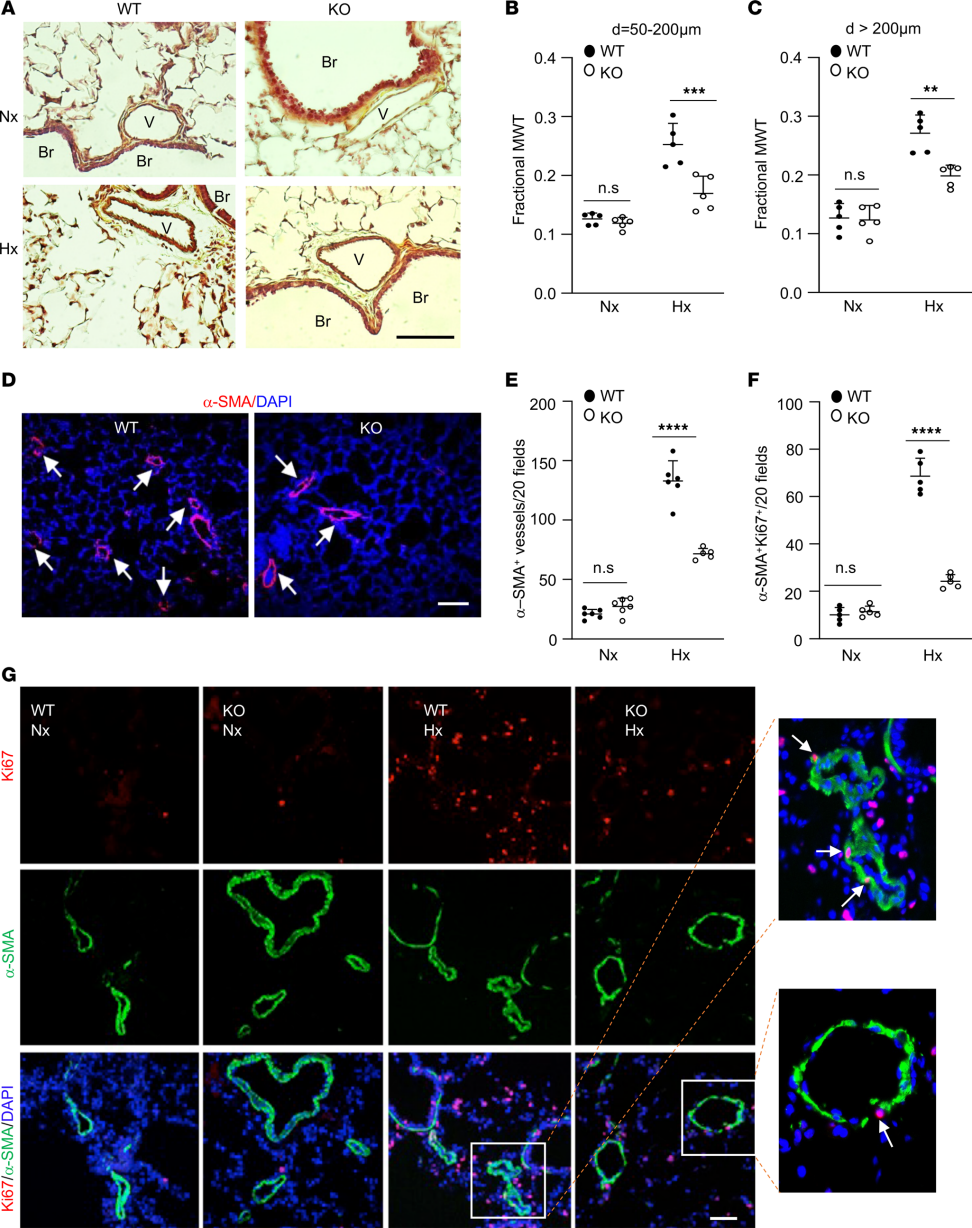
Reduced pulmonary vascular remodeling in hypoxic KO mice compared with hypoxic WT mice. (**A**) Representative micrographs of Russell-Movat pentachrome staining of mouse lung sections. Scale bar, 50 μm. Br, bronchiole; V, vessel. (**B** and **C**) Quantification of the average of pulmonary vessel wall thickness of different diameter (d) sizes of vessels. *N* = 5/group. MWT, media wall thickness. (**D**) Representative micrographs of anti–α-SMA staining of hypoxic WT and hypoxic KO lung sections showing reduced number of muscularized distal pulmonary vessels in hypoxic KO lungs compared with hypoxic WT lungs. Nuclei were counterstained with DAPI (blue). Arrows point to muscularized vessels. Scale bar, 50 μm. (**E**) Quantification of muscularized distal pulmonary vessels. The total number of α-SMA-positive distal pulmonary vessels (d ≤ 50 μm) of 20× original magnification fields of each section was used for each mouse. *n* = 20 fields of each mouse section, 5–6 mice for each group. (**F** and **G**) Quantification of smooth muscle cell (SMC) proliferation in mouse lungs. *N* = 5/group. Representative micrographs of immunostaining of mouse lung sections were shown (**G**). Mouse lungs were collected from 3.5-month-old mice under normoxia or 1-week hypoxia for sectioning and immunostaining with anti-Ki67 (red) to identify proliferative cells. SMCs were immunostained with anti–α-SMA (green). Nuclei were counterstained with DAPI (blue). Scale bar, 50 μm. Data are shown as means + SD. **, *P* < 0.01; ***, *P* < 0.001; ****, *P* < 0.0001. Two-way ANOVA with Tukey’s multiple comparisons test (**B**, **C**, **E**, and **F**).

Next, we performed pulmonary pathology examination by Russell-Movat pentachrome staining and anti–α-smooth muscle actin (α-SMA) immunofluorescence staining of these lung tissues. Russell-Movat pentachrome staining showed that hypoxia-induced increase in pulmonary vessel media wall thickness in WT mice was attenuated in KO mice (Figure 2, A–C). The number of muscularized distal pulmonary arterioles shown as α–smooth muscle actin–positive (α-SMA–positive) staining was also significantly reduced in hypoxic KO mice compared with hypoxic WT mice (Figure 2, D and E). To determine whether Gcn2 mediates pulmonary vascular EC and SMC proliferation, we assessed cell proliferation by anti-Ki67 immunostaining. After 1-week hypoxia exposure, WT mice showed a 6-fold increase in the number of proliferating SMCs (i.e., Ki67^+^α-SMA^+^ cells) (Figure 2, F and G) and a 15-fold increase of Ki67^+^CD31^+^ cells (proliferating ECs) (Supplemental Figure 4), respectively, compared with those under normoxia condition. However, hypoxic KO mice exhibited only a 2-fold increase in the number of proliferating SMCs and 4-fold increase of proliferating ECs (Figure 2, F and G, and Supplemental Figure 4). Together, these data suggest that GCN2 activation by hypoxia contributes to pulmonary vascular remodeling in hypoxic WT mice.

### GCN2 is phosphorylated and activated in response to hypoxia.

**Figure 3:**
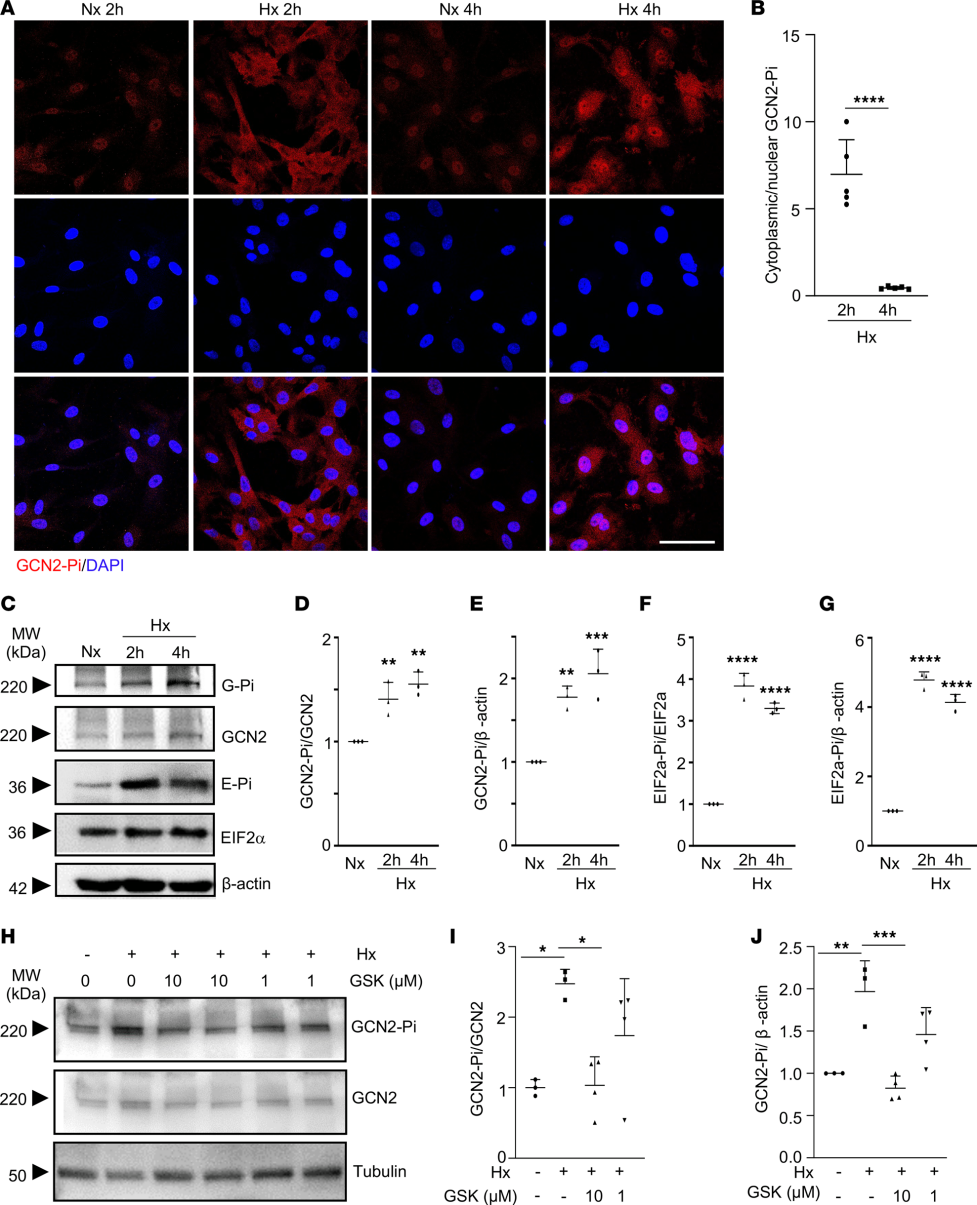
Hypoxia induces GCN2 phosphorylation and activation. (**A**) Representative micrographs of anti–phospho-GCN2 immunostaining showing GCN2 hyperphosphorylation by hypoxia challenge in primary cultures of HLMVECs. Fixed cells at indicated times after hypoxia (1% O_2_) challenge (Hx) or normoxia (Nx) were immunostained with anti-Thr899 phospho-GCN2 (red). Nuclei were counterstained with DAPI (blue). (**B**) Quantification of cytoplasmic over nuclear GCN2-Pi at 2-hour and 4-hour hypoxia exposure. *N* = 5/group. (**C**–**G**) Western blotting demonstrating hypoxia-induced GCN2 phosphorylation and activation. HLMVECs were lysed for Western blotting with anti-Thr899 phospho-GCN2 (GCN2-Pi) antibody and anti-Ser51 phospho-EIF2α (EIF2α-Pi) antibody. Total GCN2 and EIF2α levels were assessed by anti-GCN2 antibody and anti-EIF2α antibody, respectively, while anti–β-actin was used as a loading control (**C**). The band intensities of phospho-GCN2 (GCN2-Pi) and phospho-EIF2α (EIF2α-Pi) were quantified (**D**–**G**). (**H**–**J**) Western blotting demonstrating hypoxia-induced GCN2 phosphorylation was inhibited by PDK1 inhibitor (GSK, GSK2334470) treatment in a dose-dependent manner. HLMVECs in complete growth medium were treated with GSK2334470 at 10 or 1 μM or control vehicle under 2-hour hypoxia challenge (1% O_2_). Then cells were lysed for Western blotting with anti-Thr899 phospho-GCN2 (GCN2-Pi) antibody. Total GCN2 levels were assessed by anti-GCN2 antibody while anti-tubulin was used as a loading control (**H**). The band intensities of phospho-GCN2 were quantified (**I** and **J**). *N* = 3 repeat studies. Data are shown as means + SD. *, *P* < 0.05; **, *P* < 0.01; ***, *P* < 0.001; ****, *P* < 0.0001. Unpaired 2-tailed *t* test (**B**); 1-way ANOVA with Dunnett’s multiple comparisons test (**D**–**G**), with Holm-Šídák multiple comparisons test (**I**), or with Tukey’s multiple comparisons test (**J**).

To determine if GCN2 is activated in response to hypoxia in vitro, we challenged primary culture of human lung microvascular endothelial cells (HLMVECs) with hypoxia (1% O_2_). GCN2 was highly activated, evident by immunofluorescence staining of Thr899 hyperphosphorylation in hypoxic HLMVECs compared with normoxic cells (Figure 3A). It was also observed that GCN2 was activated mainly in the cytosol in response to hypoxia within 2 hours of exposure and continued to be activated but concentrated in the nucleus at 4 hours of hypoxia exposure (Figure 3B). Western blotting also verified the increase of GCN2 activity by hypoxia, evident by Thr899 phosphorylation in GCN2 and hyperphosphorylation of its downstream target EIF2α at Ser51 site (Figure 3, C–G). Given that Gcn2 can be phosphorylated by yeast Pkh1 (the ortholog of mammalian PDK1) in vitro (37) and that PDK1 can be induced by hypoxia (38), we next determined whether hypoxia-induced GCN2 phosphorylation was mediated by PDK1. Hypoxic HLMVECs were treated with PDK1-specific inhibitor GSK233470 (39). Western blotting demonstrated that hypoxia-induced GCN2 phosphorylation was inhibited by PDK1 inhibitor GSK 2334470 treatment in a dose-dependent manner (Figure 3, H–J). These data demonstrated that GCN2 is phosphorylated and activated by PDK1 in response to hypoxia in ECs.

### Hypoxia-induced Edn1 expression is suppressed by Gcn2 deficiency.

**Figure 4:**
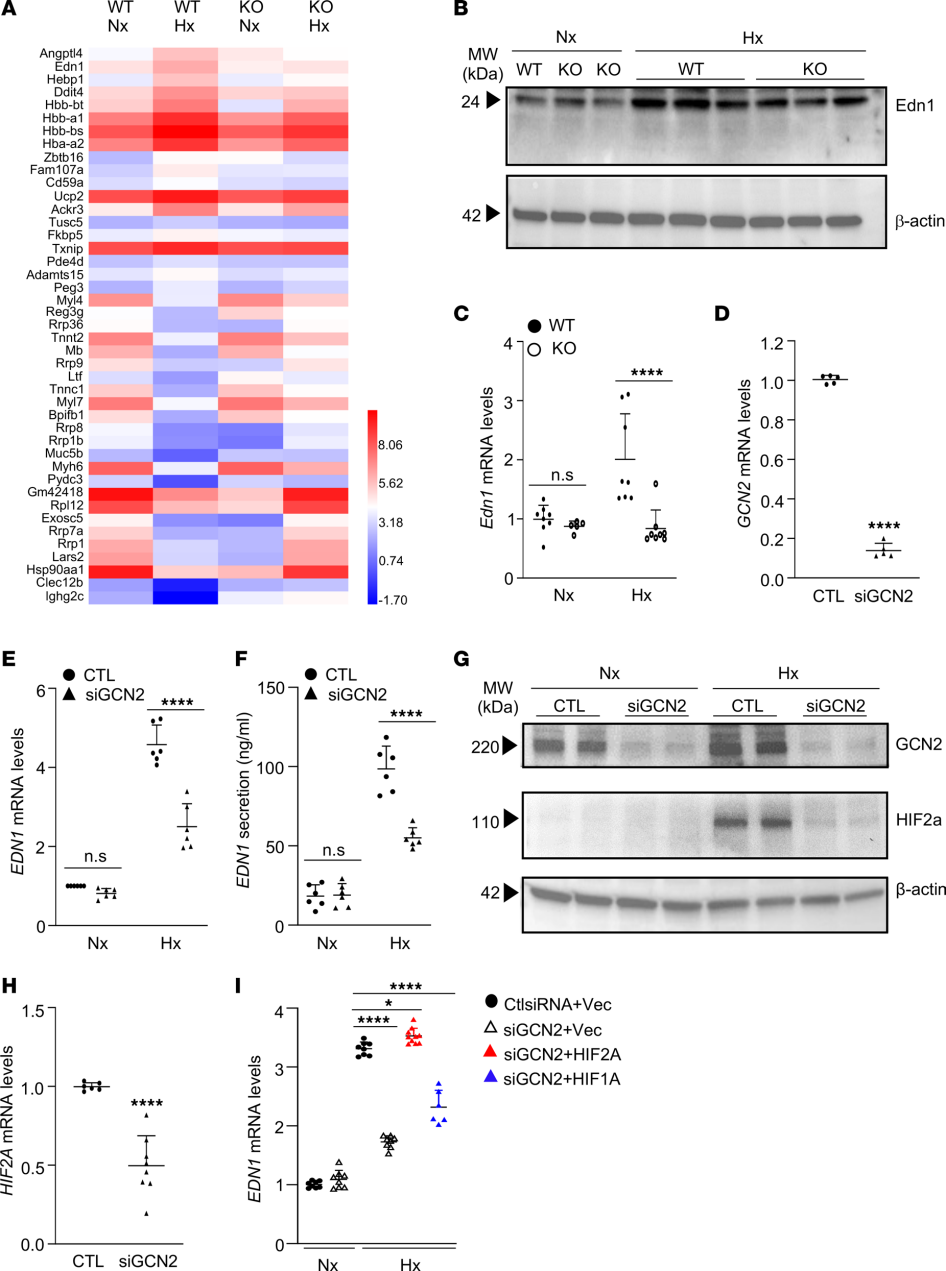
Hypoxia induces Edn1 expression through GCN2 in both mouse lungs and human lung ECs. (**A**) Representative heatmap of RNA-sequencing analysis of mouse lung tissues (*n* = 4 mice combined per group). Nx, normoxia; Hx, hypoxia. (**B**) Western blotting demonstrating Edn1 protein levels were upregulated in lung tissues of hypoxic WT mice, which were significantly reduced in hypoxic KO mice. (**C**) Quantitative RT-PCR analysis verifying *Edn1* mRNA upregulation in WT hypoxia mouse lungs but not in KO hypoxia mouse lungs. *N* = 5–8/group. (**D**) Quantitative RT-PCR analysis demonstrating GCN2 siRNA–mediated (siGCN2-mediated) knockdown of GCN2 in HLMVECs. *N* = 5/group. CTL, control siRNA. (**E**) Quantitative RT-PCR analysis demonstrating hypoxia-induced EDN1 mRNA expression was mediated by GCN2 in HLMVECs. *N* = 6/group. (**F**) ELISA of EDN1 secreted in culture medium demonstrating hypoxia-induced EDN1 protein expression was reduced by GCN2 silencing in HLMVECs. *N* = 6/group. (**G**) Western blotting confirmation of reduced HIF-2α expression in GCN2-deficient HLMVECs compared with control cells under hypoxia exposure. (**H**) Quantitative RT-PCR analysis demonstrating inhibited *HIF2A* mRNA expression in GCN2-deficient HLMVECs. Control *n* = 6, siGCN2 *n* = 8. (**I**) Quantitative RT-PCR analysis demonstrating GCN2 mediated hypoxia-induced EDN1 expression through HIF-2α rather than HIF-1α. HLMVECs were transfected with either siGCN2 or control siRNA (CtlsiRNA), and *HIF1A*, *HIF2A*, or vector plasmid, and then challenged with hypoxia or maintained in normoxia. At 48 hours after hypoxia challenge or normoxia, the cells were lysed for RNA isolation for quantitative RT-PCR analysis. *N* = 6–10/group. Data are shown as means + SD. *, *P* < 0.05; ****, *P* < 0.0001. Unpaired 2-tailed *t* test (**D** and **H**); 2-way ANOVA with Tukey’s multiple comparisons test (**C**, **E**, and **F**); 1-way ANOVA with Tukey’s multiple comparisons test (**I**).

To determine the molecular mechanism of GCN2 in regulating PH and vascular remodeling, we performed whole-transcriptome RNA-sequencing analysis of both WT and KO mouse lung tissues subjected to normoxia and hypoxia conditions (Figure 4A). RNA-sequencing data analysis showed that upregulated genes in WT lungs with hypoxia challenge, such as *Angptl4*, *Edn1*, *CD59a*, *Tusc5*, *Fkbp5*, and *Pde4d*, were completely normalized in KO lungs with hypoxia challenge, and downregulated genes in WT lungs with hypoxia challenge, such as *Lars2*, *Exosc5*, *Muc5b*, and some ribosome protein genes (*Rrp*s), were completely normalized in KO lungs with hypoxia challenge (Table 1 and Table 2). Edn1, a potent vasoconstrictor in the pathogenesis of PAH (40–43), is ranked in the top 2 genes of the upregulated gene list, suggesting Edn1 as a potential downstream target gene of GCN2 in regulating PH. Western blotting (Figure 4B) and quantitative RT-PCR analysis (Figure 4C) verified increased expression of Edn1 in WT mouse lungs with hypoxia compared with those with normoxia, whereas expression was significantly reduced in KO mouse lungs with hypoxia.

To further determine the effect of GCN2 deficiency on Edn1 expression, which is predominantly expressed in ECs, we knocked down GCN2 with *GCN2* siRNA in HLMVECs and exposed the cells to hypoxia. Quantitative RT-PCR analysis demonstrated a 90% knockdown efficiency of GCN2 (Figure 4D). As expected, hypoxia (1% O_2_) exposure induced a ~4-fold increase in *EDN1* mRNA expression and secreted EDN1 protein in control cells, while the increases were markedly reduced in GCN2-deficient cells (Figure 4, E and F), demonstrating that hypoxia induces EDN1 expression through GCN2 signaling.

Since EDN1 is a downstream target of hypoxia-inducible factor (HIF) 1 (44, 45), we further determined whether GCN2 regulates EDN1 through HIFs. Western blotting demonstrated that hypoxia-induced increases of HIF-2α protein levels were inhibited in GCN2-deficient HLMVECs (Figure 4G). Quantitative RT-PCR analysis also showed reduced mRNA levels of *HIF2A* in hypoxic GCN2-deficient HLMVECs compared with control cells (Figure 4H). However, hypoxia-induced increases of HIF-1α protein levels were largely not reduced in GCN2-deficient HLMVECs (Supplemental Figure 5A). To determine whether HIF-1α or HIF-2α overexpression can rescue EDN1 expression in GCN2-deficient HLMVECs, GCN2 siRNA or control siRNA and *HIF2A* plasmid, *HIF1A* plasmid, or vector plasmid were cotransfected to HLMVECs, and the cells were then exposed to hypoxia. Quantitative RT-PCR analysis showed that hypoxia-induced *EDN1* expression was inhibited by GCN2 deficiency, which was fully rescued by *HIF2A* plasmid–mediated overexpression of HIF-2α. However, HIF-1α overexpression only partially rescued EDN1 expression (Figure 4I and Supplemental Figure 5, B and C), demonstrating that GCN2 regulates EDN1 expression by regulating HIF-2α expression rather than HIF-1α.

Since GCN2 regulates EDN1 expression in ECs in response to hypoxia, we next determined if endothelial GCN2 is involved in EC-induced pulmonary arterial vascular smooth muscle cell (PASMC) proliferation by secreting EDN1. Conditioned media collected from GCN2-deficient HLMVECs or control cells, which were challenged with hypoxia or normoxia, were added to primary cultures of PASMCs under normoxia. Conditioned media from hypoxic control HLMVECs induced a 2-fold increase of PASMC proliferation compared with that from normoxic control HLMVECs, whereas conditioned medium from hypoxic, GCN2-deficient HLMVECs induced only a negligible increase of PASMC proliferation compared with normoxic cells (Supplemental Figure 6).

### Restored endothelial Edn1 expression in KO mice partially reverses the reduced PH phenotype.

**Figure 5:**
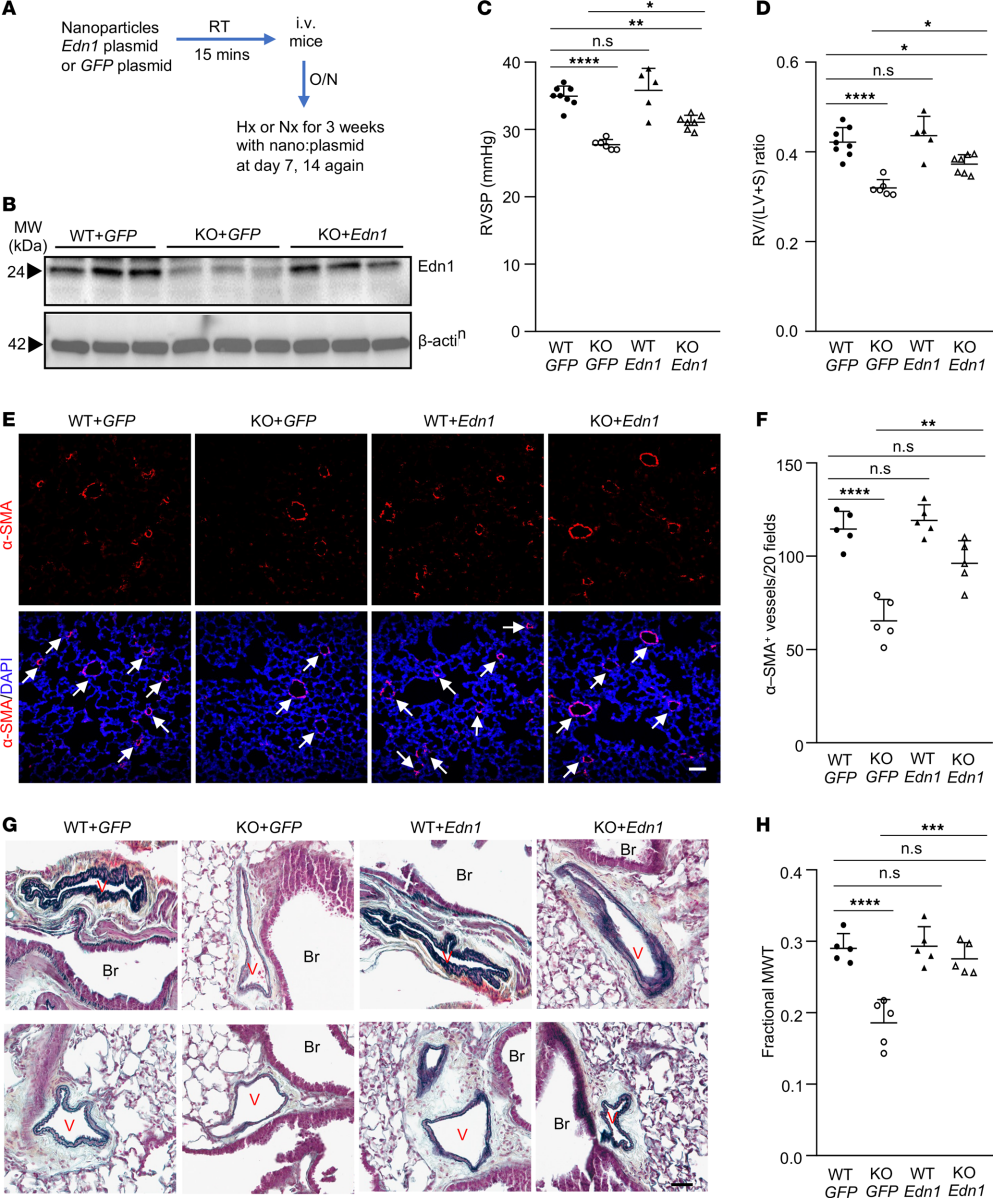
Restored expression of endothelial Edn1 in Gcn2-deficient mice partially reverses the reduced PH phenotype. (**A**) Diagram showing the experimental procedure to restore *Edn1* expression selectively in ECs of WT and *Gcn2*-deficient mice. Mixture of nanoparticles/plasmid DNA expressing *Edn1* or *GFP* (control) under the control of *CDH5* promoter was administered retro-orbitally to 11-week-old KO mice and WT mice. After overnight (16 hours), the mice were subjected to hypoxia. Nanoparticles/plasmid DNA mixtures were administered weekly for 3 doses total, and each mouse received 30 μg plasmid DNA each time. (**B**) Western blotting demonstrating restored Edn1 expression in ECs of KO mice with *Edn1* plasmid administration compared with WT and KO mice with *GFP* plasmid. Lung ECs were isolated for Western blotting. (**C**) RVSP measurement showing reduced PH in KO mice with *GFP* plasmid was partially reversed in KO mice with *Edn1* plasmid. (**D**) RV/(LV+S) ratio also showing reduced RV hypertrophy in KO mice with *GFP* plasmid was partially rescued with restored *Edn1* expression. *N* = 5–8/group. (**E**) Representative micrographs of anti–α-SMA staining of lung sections showing reduced number of muscularized distal pulmonary vessels in hypoxic KO+*GFP* lungs was partially reversed in hypoxic KO+*Edn1* lungs. Nuclei were counterstained with DAPI (blue). Arrows point to muscularized vessels. (**F**) Quantification of the number of muscularized distal pulmonary vessels. *N* = 5/group. (**G**) Representative micrographs of Russell-Movat pentachrome staining of mouse lung sections. Br, bronchiole; V, vessel. (**H**) Quantification of pulmonary vessel media wall thickness. *N* = 5/group. Scale bars, 50 μm. Data are shown as means + SD. *, *P* < 0.05; **, *P* < 0.01; ***, *P* < 0.001; ****, *P* < 0.0001. One-way ANOVA with Tukey’s multiple comparisons test (**C**, **D**, **F**, and **H**).

We next performed a rescue study in vivo employing the endothelium-targeted nanoparticle delivery of a gene (35) to determine if restoration of endothelial Edn1 expression in KO mice can reverse the reduced phenotype of PH in response to hypoxia. Mixture of EndoNP1 nanoparticles/plasmid DNA expressing mouse *Edn1* under the control of human *CDH5* promoter, or *GFP* plasmid (as a control), was administered retro-orbitally into WT and KO mice weekly (Figure 5A). After 3 weeks of exposure to hypoxia (10% O_2_), mouse lung ECs were isolated for Western blotting. Edn1 was highly expressed in KO mice with *Edn1* plasmid administration compared with those with *GFP* control plasmid DNA (Figure 5B). Hemodynamic measurements demonstrated that hypoxia-induced increase of RVSP and RV/(LV+S) ratio in WT mice with *GFP* plasmid DNA were not further elevated in WT mice with *Edn1* plasmid DNA; however, reduced RVSP and RV/(LV+S) ratio seen in hypoxic KO mice with GFP plasmid DNA compared with hypoxic WT mice were partially reversed in *Edn1* plasmid DNA-administered hypoxic KO mice (Figure 5, C and D). Under normoxia condition, both WT and KO mice with *Edn1* plasmid DNA administration exhibited mild increases in RVSP and RV/(LV+S) ratio compared with those with GFP plasmid DNA (Supplemental Figure 7). α-SMA immunostaining and histological assessment showed that the reduced number of muscularized distal pulmonary arterioles and decreased pulmonary arterial medium wall thickness in KO mice with *GFP* plasmid DNA compared with hypoxic WT mice were partially rescued in *Edn1* plasmid DNA–administered KO mice (Figure 5, E–H). Taken together, these data demonstrated that decreased endothelial Edn1 expression in KO mice plays a causal role in attenuating hypoxia-induced PH.

### Pharmacological inhibition of GCN2 kinase suppresses PAH in monocrotaline rats.

**Figure 6:**
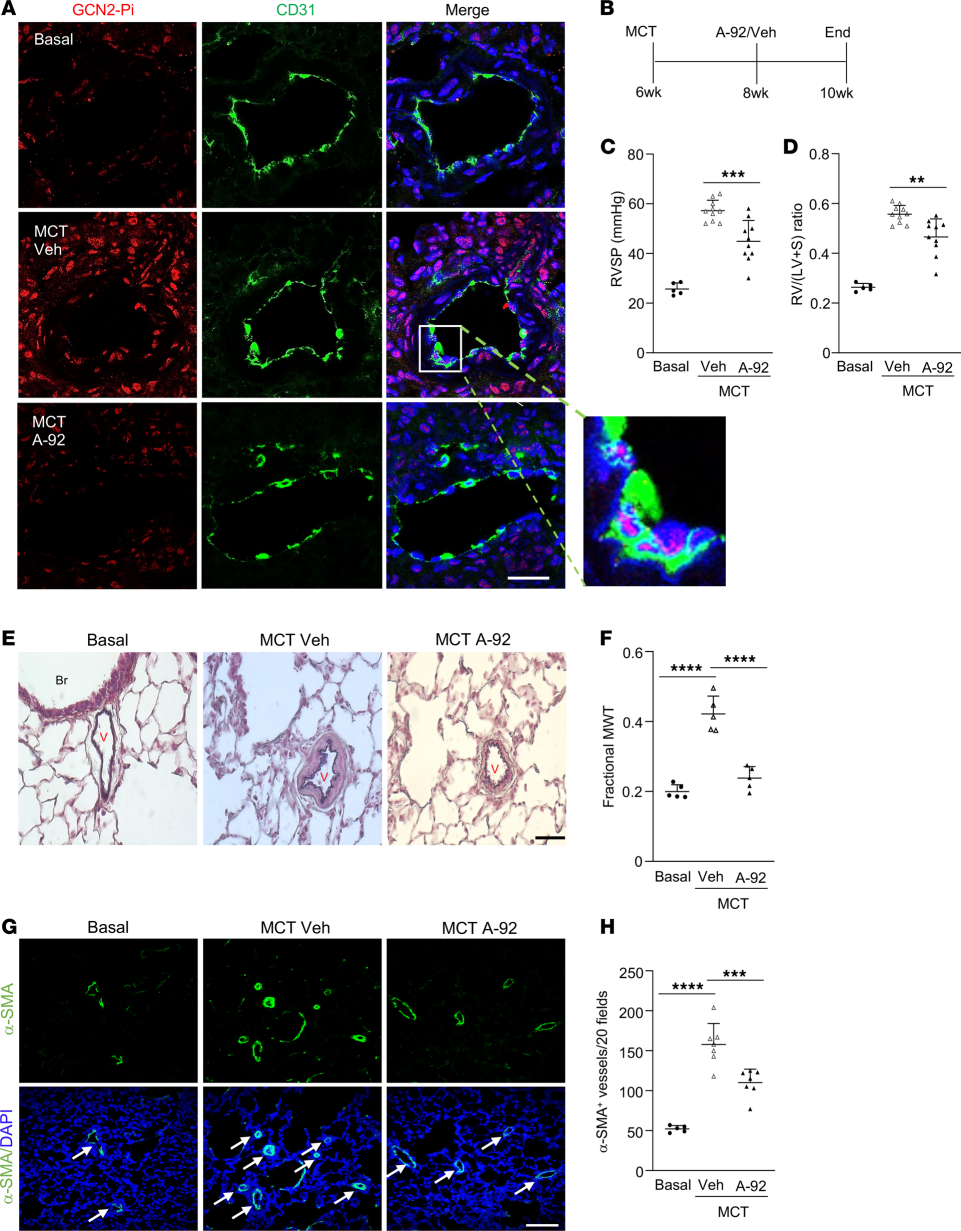
Pharmacological inhibition of GCN2 kinase attenuates MCT-induced PAH in rats. (**A**) Representative micrographs of anti–phospho-GCN2 immunostaining of rat lung sections demonstrating prominent Thr898 phosphorylation-GCN2 (GCN2-Pi) in pulmonary vascular ECs in vehicle-treated MCT rats, which was inhibited in A-92–treated MCT rat lungs. Lung tissue cryosections from basal control rats, vehicle-treated MCT rats (4 weeks), or compound A-92–treated MCT rats were immunostained with anti-Thr898 phospho-GCN2 antibody (red). ECs were immunostained with anti-CD31 (green), and nuclei were counterstained with DAPI (blue). Scale bar, 50 μm. (**B**) Graphical presentation of the experimental procedure. A-92 (0.5 mg/kg, i.p. daily) was administered to rats at 2 weeks after MCT. (**C**) RVSP measurement showing a marked increase of RVSP in MCT rats treated with vehicle compared with basal rats, which was reduced in A-92–treated MCT rats. (**D**) RV hypertrophy evident by increased RV/(LV+S) ratio seen in vehicle MCT rats was reduced in A-92 MCT rats. Basal *n* = 5, MCT veh *n* = 10, MCT A92 *n* = 10. (**E**) Representative micrographs of Russell-Movat pentachrome staining of rat lung sections showing attenuated vessel wall thickening. Br, bronchiole; V, vessel. (**F**) Quantification of average pulmonary vessel wall thickness. *N* = 5/group. MWT, media wall thickness. (**G**) Representative micrographs of anti–α-SMA (green) staining of basal rat, MCT vehicle rat, and A-92–treated MCT rat lung sections showing reduced number of muscularized distal pulmonary vessels in A-92–treated MCT rat lungs. Nuclei were counterstained with DAPI (blue). Arrows point to muscularized distal pulmonary vessels. (**H**) Quantification of muscularized distal pulmonary vessels. The total number of α-SMA–positive distal pulmonary vessels (d ≤ 50 μm) of 20× original magnification fields of each section was used for each rat. *N* = 5–7/group. Arrows point to muscularized vessels. Data are shown as means + SD. Scale bars, 50 μm. **, *P* < 0.01; ***, *P* < 0.001; ****, *P* < 0.0001. One-way ANOVA with Tukey’s multiple comparisons test (**C**, **D**, **F**, and **H**).

To determine if GCN2 is a potential therapeutic target for PAH treatment, we first conducted immunofluorescence staining with anti-Thr899 phospho-GCN2 in rat lungs challenged with monocrotaline (MCT). GCN2 was hyperphosphorylated in vascular ECs and SMCs in MCT rat lungs in contrast with naive control rats, suggesting GCN2 hyperactivation in response to MCT challenge (Figure 6A and Supplemental Figure 8A). We next determined if pharmacological inhibition of GCN2 kinase could inhibit PAH in MCT rats. At 2 weeks after MCT challenge, the rats were treated with either GCN2 inhibitor A-92 (46) (Supplemental Figure 8B) or vehicle (Figure 6B). Western blotting and immunofluorescence staining demonstrated MCT-induced GCN2 activation in rat lung tissue was inhibited by A-92 treatment (Figure 6A and Supplemental Figure 8, A and C). Accordingly, MCT-induced increases in RVSP and RV/(LV+S) ratio were significantly reduced in A-92–treated rats compared with vehicle-treated ones (Figure 6, C and D, and Supplemental Figure 8, D and E). A-92 treatment attenuated pulmonary vascular remodeling, including reduced media wall thickening and the number of muscularized distal pulmonary vessels in MCT rats (Figure 6, E–H). A-92 treatment also reduced the increases of vascular SMC and EC proliferation in MCT rats (Supplemental Figures 9 and 10). Together, these data suggest that inhibition of GCN2 activation suppresses pulmonary vascular remodeling and PAH development, supporting GCN2 as a potential therapeutic target for PAH treatment.

### GCN2 is highly activated in pulmonary vascular ECs of patients with IPAH without marked changes of protein expression.

**Figure 7:**
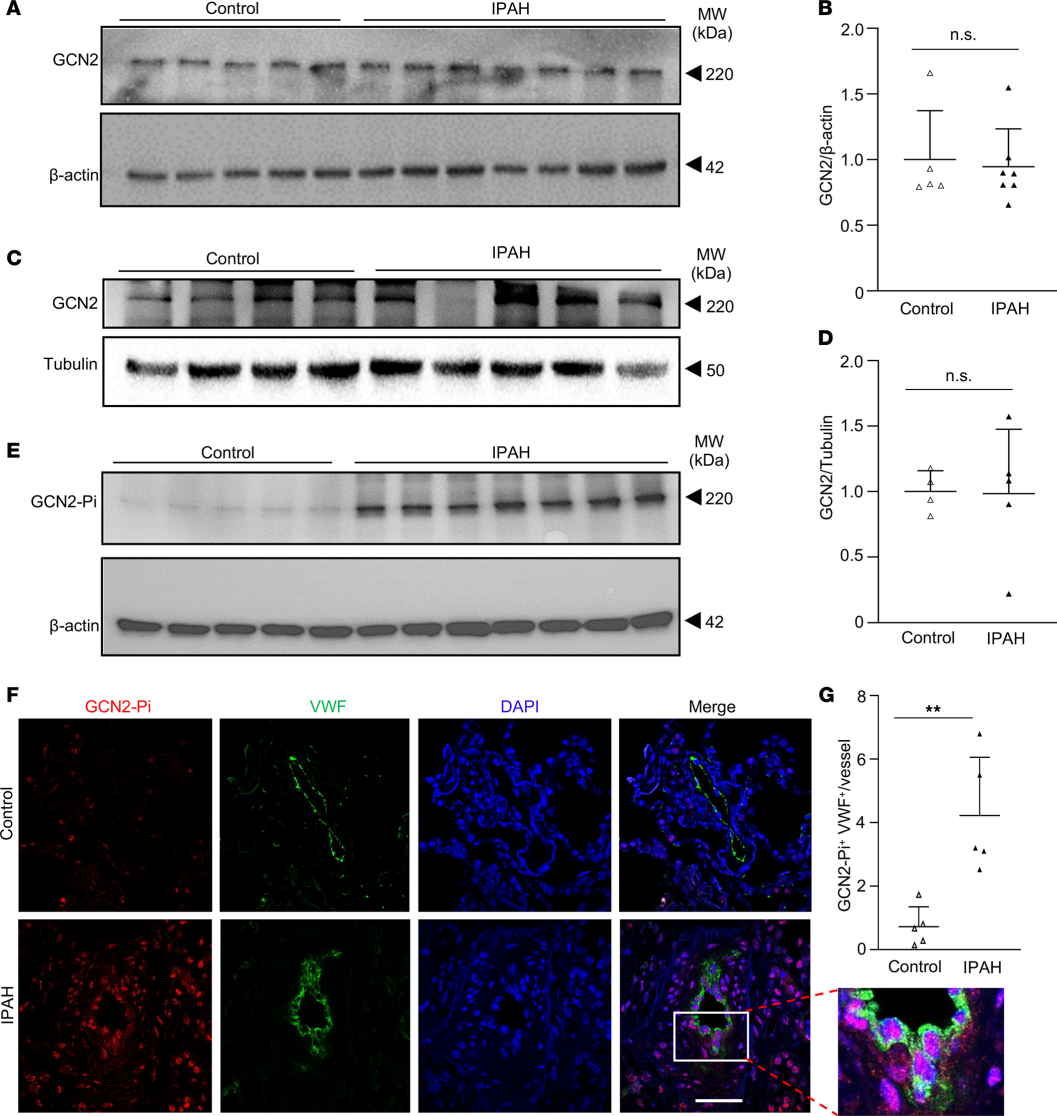
Prominent GCN2 phosphorylation/activation in ECs of pulmonary vascular lesions of patients with IPAH. (**A** and **B**) Western blotting of anti-GCN2 and quantification demonstrating no difference in GCN2 total protein expression in whole lung tissue lysates of patients with IPAH compared with normal donors. Control *n* = 5, patients with IPAH *n* = 7. (**C** and **D**) Western blotting of anti-GCN2 and quantification demonstrating no difference in GCN2 total protein expression in pulmonary arterial ECs of patients with IPAH compared with healthy donors. Control *n* = 4, patients with IPAH *n* = 5. (**E**) Western blotting demonstrating extensive GCN2 phosphorylation in IPAH patient lung tissues but minimal in control donor lung tissues. (**F** and **G**) Prominent GCN2 phosphorylation in ECs of pulmonary vascular lesions of patients with IPAH but not in normal donor lungs. Formalin-fixed lung sections from 5 patients with IPAH and 5 non-PAH donors were immunostained with anti-Thr899 phospho-GCN2 (GCN2-Pi) (red) and anti-vWF (green). Representative micrographs of immunofluorescence staining were shown (**F**). The average number of GCN2-Pi^+^ ECs in each vessel was quantified (*n* = 5 samples/group, 15 vessels/sample) (**G**). Data are shown as means + SD. Scale bar, 50 μm. **, *P* < 0.01. Mann-Whitney *U* test (**B**). Unpaired 2-tailed *t* test (**D** and **G**).

To address the clinical relevance of GCN2 activation in the pathogenesis of PAH, we first quantified GCN2 mRNA expression in lung tissues from healthy donors and patients with IPAH (Supplemental Table 1) by quantitative RT-PCR analysis. Although GCN2 mRNA expression varied among individuals in both the control donor group and the IPAH patient group, there was no marked difference in the mRNA levels between the 2 groups (Supplemental Figure 11). Western blotting also revealed similar GCN2 protein levels in lungs of donors and patients with IPAH (Figure 7, A and B). Similarly, there was no marked difference in GCN2 protein levels in pulmonary arterial ECs of donors and patients with IPAH (Figure 7, C and D). We next determined whether GCN2 phosphorylation and activation differs between donors and patients. Western blotting analysis showed markedly increased phosphorylation of GCN2 in whole lung tissues of patients with IPAH compared with control donors (Figure 7E). Lung sections were immunostained with anti-Thr899 phospho-GCN2 and anti-VWF to assess GCN2 activation in pulmonary vascular ECs. The number of GCN2-phosphorylated vascular ECs in IPAH patient lungs was about 5-fold higher than the number of those in donor lungs, suggesting highly activated GCN2 in pulmonary vasculature of patients with IPAH (Figure 7, F and G). These data support the concept that GCN2 activation in ECs contributes to pulmonary vascular remodeling and PAH development in patients.

## Discussion

The present study supports an important role of GCN2 kinase activation in mediating pulmonary vascular remodeling and PAH development. We observed no spontaneous PVOD and PH in KO mice. However, loss of GCN2 kinase domain attenuated hypoxia-induced pulmonary vascular remodeling and PH in mice. Mechanistic studies demonstrated that GCN2 activation in HLMVECs mediated hypoxia-induced EDN1 expression via HIF-2α. Restored Edn1 expression in mouse lung ECs partially rescued the reduced PH phenotype of KO mice in response to hypoxia. Moreover, inhibition of GCN2 kinase activity through A-92 treatment attenuated MCT-induced PAH and pulmonary vascular remodeling in rats. In IPAH lungs, GCN2 phosphorylation was markedly increased in pulmonary vascular ECs without marked changes of GCN2 expression. Together, these data demonstrate the requisite role of GCN2 kinase activation in mediating pulmonary vascular remodeling and PAH development. Thus, targeting GCN2 signaling is a potential therapeutic strategy for treatment of PAH without GCN2 loss-of-function mutations.

Published studies have demonstrated a strong relationship of loss-of-function mutations of *EIF2AK4* and PVOD development in patients. However, loss of GCN2 kinase domain in mice did not induce spontaneous PVOD, which is consistent with a previous observation that the KO mice are viable, are fertile, and do not exhibit an obvious phenotype under standard housing conditions (47). Given that the KO mice used in these studies have genetic deletion of exon 12 of the *Eif2ak4* gene encoding amino acids 606–648, an essential region for its kinase activity (14), it is possible that the N-terminal 605 amino acids may retain some of the GCN2 functional domains, which are sufficient to inhibit PVOD development. Among 41 reported variants of *EIF2AK4* gene associated with classic PAH (i.e., non-PVOD PAH), none of them are located in exon 12 of the gene (48); while among 32 reported variants of *EIF2AK4* associated with PVOD or pulmonary capillary hemangiomatosis, only 2 of them are located in exon 12, including 1 missense variant at amino acid 643 (31), suggesting that most of the reported mutations of *EIF2AK4* associated with PAH or PVOD are not related to exon 12 in the essential kinase domain. This may explain the observation that deletion of exon 12 in the GCN2 kinase domain does not induce spontaneous PAH or PVOD. Comparing all the reported variants of *EIF2AK4* between patients with classic PAH and PVOD, only 3 variants are shared (48), indicating that mutations in different functional domains of GCN2 may play distinct roles in the development of classic PAH and PVOD. It is also possible that a second “hit” coupled with loss of GCN2 is required for PVOD development.

Our studies show GCN2 hyperphosphorylation of Thr898 at the kinase domain, which indicates GCN2 activation (21–25) in pulmonary vascular ECs and SMCs of hypoxic WT mice. We also observed rapid and extensive phosphorylation of GCN2 in cultured HLMVECs in response to hypoxia and GCN2 is required for hypoxia-induced vascular cell proliferation in mice. Thus, hypoxia-induced activation of GCN2 plays a causal role in mediating pulmonary vascular remodeling and PH development. Surprisingly, we also observed an extensive GCN2 phosphorylation in pulmonary vascular ECs in patients with IPAH, demonstrating the clinical relevance of GCN2 activation in the pathogenesis of PAH. There are contradictory reports about GCN2 protein levels in IPAH lungs. One report shows diminished GCN2 protein levels in IPAH lungs and MCT-treated rat lungs, indicating loss of GCN2 induces PAH (49). Another report, however, demonstrates similar GCN2 protein levels in IPAH and control lungs (50). Our study here shows no marked difference in GCN2 expression, at both mRNA and protein levels, in whole lung tissues as well as pulmonary vascular ECs between IPAH patient lungs and control lungs. Furthermore, our study provides unequivocal evidence of extensive GCN2 phosphorylation in pulmonary vascular lesions of patients with IPAH, especially in vascular ECs. These data demonstrate that GCN2 activation, rather than GCN2 expression change, plays a crucial role in pulmonary vascular remodeling and PAH development in patients.

In response to amino acid starvation, GCN2 is activated by the binding of uncharged tRNAs accumulated in amino acid–starved cells to the HisRS-related domain of GCN2 (17–19), inducing conformational changes (20, 21) and autophosphorylation. Since hypoxia induces GCN2 phosphorylation at Thr899 in the kinase domain indicative of GCN2 activation in HLMVECs with complete growth medium as early as 2-hour exposure, hypoxia-induced activation of GCN2 is independent of the canonical uncharged tRNA binding. Consistently, previous studies also show that GCN2 can be activated independent of tRNA binding; for example, mutations at or flanking the protein kinase domain of GCN2 cause constitutive activation (25). GCN2 can be activated by hypoxia in mouse embryonic fibroblast cells (28); however, the potential mechanism leading to GCN2 activation is unclear. In our study, we have demonstrated that hypoxia activated and phosphorylated GCN2 through PDK1. Since we also observed GCN2 hyperphosphorylation in pulmonary vascular SMCs in MCT rats and patients with IPAH, it is possible that GCN2 can also be activated by other PAH-causing factors, such as growth factors or inflammatory mediators (51), to promote pulmonary vascular remodeling and PAH development. Future studies are warranted to delineate the molecular mechanisms leading to GCN2 activation in different cell types including ECs and SMCs in response to various PAH-causing factors and the cell-specific role of GCN2 activation in pulmonary vascular remodeling and PAH development.

EDN1, an EC-derived factor, plays an important role in the pathogenesis of PAH in increasing vasoconstriction and cell proliferation. EDN1 exerts vasoconstrictor and mitogenic effects by binding to the 2 distinct receptors on vascular SMCs, making it the main therapeutic target in the treatment of PAH (41–43). Our study demonstrates the role of GCN2 in regulating EDN1 expression in the hypoxia PH mouse model and in cultured HLMVECs in response to hypoxia. Our data have shown that GCN2 deficiency inhibited hypoxia-induced EDN1 expression in HLMVECs, which was completely rescued by HIF-2α overexpression while only partially rescued by HIF-1α overexpression when the same amount of HIF plasmid DNA was transfected to HLMVECs. These data demonstrate that GCN2 regulates hypoxia-induced EDN1 expression through HIF-2α rather than HIF-1α as previously reported (44, 45). Recent studies also support the role of HIF-2α in mediating EDN1 expression in mouse lungs and HLMVECs (52, 53). Furthermore, we employed the endothelium-targeted nanoparticles to deliver *Edn1* gene specifically into ECs in KO mice and observed partially reversed PH phenotype in these mutant mice, demonstrating the casual role of the PDK1/GCN2/HIF-2α/EDN1 signaling axis in the mechanisms of pulmonary vascular remodeling and PH development in response to chronic hypoxia. The partial rescue effect of *Edn1* overexpression in mouse lung ECs suggests that GCN2 may regulate expression of other PH-causing factors in ECs or that GCN2 activation in other cell types such as SMCs also contributes to pulmonary vascular remodeling and PAH development.

In conclusion, our data have demonstrated that disruption of the GCN2 kinase domain did not induce PVOD in mice but unexpectedly inhibited pulmonary vascular remodeling and PH in response to chronic hypoxia. We found that hypoxia-induced Edn1 expression is mediated by GCN2 activation via HIF-2α expression and that restored endothelial Edn1 expression in KO mice partially reversed the reduced PH phenotype, demonstrating the important role of the PDK1/GCN2/HIF-2α/EDN1 signaling axis in the pathogenesis of pulmonary vascular remodeling and PAH. Furthermore, MCT-induced PAH and vascular remodeling was suppressed by inhibiting GCN2 activity. Importantly, we also observed extensive GCN2 phosphorylation/activation in ECs of pulmonary vascular lesions of patients with IPAH without marked changes of protein levels. Thus, targeting GCN2 kinase activation represents a promising therapeutic strategy for treatment of patients with IPAH, especially those without GCN2 loss-of-function mutations.

## Methods

### Sex as a biological variable.

Our study examined male and female animals, as well as lung tissue samples from both male and female individuals, and similar findings are reported for both sexes.

### Mice and hypoxia study.

*Eif2ak4^–/–^* (KO) mice (The Jackson Laboratory 008240) were backcrossed with C57BL/6 WT mice (The Jackson Laboratory 000664) for 5 generations to maintain C57BL/6 background. Both male and female mice were used for experiments. At the age of 11 weeks, WT and KO mice were exposed to 10% O_2_ in a hypoxia chamber (BioSpherix) for 3 weeks and then subjected to hemodynamic measurements.

### Overexpression of Edn1 in ECs in mice.

Mouse *Edn1* plasmid was obtained from GenScript (catalog OMu20519) and subcloned into pGL3 plasmid containing human *CDH5* promoter. EndoNP1 nanoparticles (MountView Therapeutics LLC, catalog EndoNP1) and 30 μg *CDH5*-*Edn1* plasmid or control GFP plasmid were mixed following manufacturer’s instructions and incubated at room temperature (RT) for 15 minutes and then administered to 11-week-old WT or KO mice. After overnight, the mice were exposed to chronic hypoxia (10%) for 3 weeks. The mixture of nanoparticles/plasmid DNA was administered weekly for 3 times total.

### Hemodynamic measurement.

RVSP was measured with 1.4F (for mice) or 3.5F (for rats) pressure transducer catheter (Millar Instruments), which was inserted into the right ventricle through right jugular vein of isoflurane-anesthetized rodents. The tracings were recorded and analyzed by AcqKnowledge software (Biopac Systems Inc.). Mouse/rat hearts were then dissected to calculate the RV/(LV+S) ratio for assessment of RV hypertrophy.

### RNA sequencing and data analysis.

After hemodynamic measurements, lung samples were collected for total RNA isolation with TRIzol (Invitrogen). RNA was further purified with RNeasy Mini Kit (QIAGEN). Each of the 4 samples from the same group were combined as 1 sample for RNA-sequencing analysis. All samples passed RNA purity before library construction through NanoDrop, agarose gel electrophoresis, and Agilent 2100 check. mRNA was purified from total RNA using poly-T oligo-attached magnetic beads. RNA libraries were prepared by using New England BioLabs library, and the final library was obtained by PCR amplification and purification of PCR products by AMPure XP beads. After library construction, we diluted the library to 1.5 ng/μL with preliminary quantitative result by Qubit 2.0 and detected the insert size by Agilent 2100. Libraries were fed into HiSeq/MiSeq machines after pooling. Raw data were sequenced on Illumina HiSeq 2500/MiSeq platform and were transformed to sequenced reads by CASAVA base recognition. Raw data were stored in FASTQ format files and submitted to National Center for Biotechnology (NCBI) Sequence Read Archive (SRA) database (BioProject PRJNA994888). The sequenced reads containing low-quality reads were filtered to get the clean reads. Reads were mapped to the reference mouse genome by TopHat 21 to accomplish the alignment, and the annotated transcripts were assembled. Read counts were used to estimate gene expression level. Genes were identified as significantly differentially expressed at 2-fold change by using the DESeq R package with adjusted *P* values < 0.05 by the Benjamini-Hochberg approach for controlling the false discovery rate. The heatmap was generated by using HemI 2.0 (Heat Map Illustrator 2.0, https://hemi.biocuckoo.org/).

### MCT-induced PAH rats and compound A-92 treatment.

Sprague-Dawley rats (Charles River Laboratories) at the age of 6 weeks (150–170 g) were challenged with MCT subcutaneously at the dose of 33 mg/kg body weight (MedChemExpress, catalog HY-N0750). Fourteen days after MCT, the rats were randomized to receive either GCN2 inhibitor compound A-92 (Axon Medchem, catalog Axon 2720) (0.5 mg/kg/d, i.p.) or vehicle treatment for 14 days. Hemodynamic measurements were then carried out.

### Primary cultures of HLMVECs.

HLMVECs from controls (Lonza, catalog cc2527) were cultured in a T75 flask in endothelial basal medium (Lonza, catalog cc-3156) supplemented with 10% FBS and EGM-2 MV Microvascular Endothelial Cell Growth Medium SingleQuots supplements and growth factors (Lonza, catalog cc-4147). HLMVECs were used between passages 4 and 7. First, to determine the effects of hypoxia on GCN2 activation, HLMVECs were seeded in 6-well plates and grown to 80% confluence in EGM-2 medium, then replaced with hypoxia-equilibrated EGM-2 medium and exposed to hypoxia (1% O_2_) or replaced with fresh EGM-2 medium under normoxia for culturing. Hypoxic conditions were achieved in an O_2_ Control In vitro Glove Box (Coy Laboratory Products, Inc), which contains an oxygen sensor monitoring oxygen levels continuously. At various hours after exposure, cells were fixed for immunofluorescence staining to assess GCN2-Thr899 phosphorylation or lysed with RIPA buffer for Western blotting to assess GCN2-Thr899 phosphorylation and EIF2α-Ser51 phosphorylation. Second, to determine the effects of hypoxia on gene expression, HLMVECs were seeded and transfected with siRNA or plasmid in 6-well plates and cultured to reach approximately 70% confluence and then starved overnight in FBS-free endothelial cell basal medium. After starvation, cells were replaced with fresh serum-free basal medium and exposed to hypoxia or normoxia for 48 hours to assess EDN1 expression level.

### Transfection with GCN2 siRNA and HIF plasmid DNA.

GCN2 siRNA was synthesized from Integrated DNA Technologies (IDT) (sense: 5′-rGrCrArArUrUrCrUrGrUrGrGrUrGrCrArUrArATT-3′, antisense: 5′-rUrUrArUrGrCrArCrCrArCrArGrArArUrUrGrCTT-3′). *HIF1A* and *HIF2A* plasmids were obtained from Addgene (catalog 18949 and catalog 18950). Control vector plasmid was generated from enzyme digestion of *HIF2A* plasmid to remove *HIF2A* gene. Transfection of siRNA or plasmid DNA was performed using Lipofectamine 3000 transfection reagent (Invitrogen, catalog L3000-008) according to the manufacturer’s protocol. Briefly, 1 μL of 50 μM siRNA or control and 10 ng of each plasmid DNA were mixed with 3.75 μL of Lipofectamine 3000 in 250 μL Opti-MEM (Gibco, Catalog 31985070) and added to each well of a 6-well plate containing 1 mL of complete growth medium for culturing for 8 hours. The cells were then cultured in complete growth medium for 24 hours followed by starvation in basal medium without FBS for hypoxia challenge.

### RNA isolation and quantitative RT-PCR analysis.

Total RNA was isolated from cultured HLMVECs with RNeasy Mini Kit including DNase I digestion (QIAGEN). Total RNA from frozen mouse lung tissues and human lung tissues was isolated with TRIzol reagents followed by clean-up with RNeasy Mini Kit including DNase I digestion. One microgram of RNA was transcribed into cDNA with high-capacity cDNA reverse transcription kits (Applied Biosystems). Quantitative RT-PCR analysis was performed on Quant Studio 6 Flex system (Life Technologies) with the Fast SYBR Green Master Mix (Applied Biosystems, catalog 4385614). Target mRNA expression was determined by the comparative cycle threshold method of relative quantitation. We used 18S rRNA gene as an internal control for analysis of expression of human genes, while cyclophilin A (*Ppia*) was used for mouse genes. The primers listed below were synthesized by IDT: *mPpia*, forward primer 5′-GGCAAATGCTGGACCAAACAC-3′, and reverse primer, 5′-TTCCTGGACCCAAAACGCTC-3′; *mGcn2*, forward primer 5′-GTTCTCCCGGTACTTCATTGAG-3′; and reverse primer, 5′-TTGCAGGGTTGATAGGGATG-3′; *mEdn1*, forward primer 5′-TTCCCAATAAGGCCACAGACC-3′, and reverse primer, 5′-TTGGGCCCTGAGT-TCTTTTCC-3′; *mPerk*, forward primer 5′-CAGGCTTTTCCATCCTCAGC-3′, and reverse primer, 5′-GGCACTCACGGAGTCGTATTT-3′; *hRNA18S*, forward primer 5′-TTCCGACC-ATAAACGATGCCGA-3′, and reverse primer, 5′-GACTTTGGTTTCCCGGAAGCTG-3′; *hGCN2*, forward primer 5′-GAAGCTGTCAGCCAGCACTA-3′, and reverse primer, 5′-GGCAAGGGAGGTCTGAAGTC-3′; *hHIF1A*, forward primer 5′-TTACAGCAGCCAGACG-ATCATG-3′, and reverse primer, 5′-TGGTCAGCTGTGGTAATCCACT-3′; *hHIF2A*, forward primer 5′-CTGATGGCCATGAACAGCATCT-3′, and reverse primer, 5′-TCCTCGAAGTT-CTGATTCCCGA-3′; *hEDN1*, forward primer 5′-GTCTACTTCTGCCACCTGGAC-3′, and reverse primer, 5′-TCCAAGGCTCTCTTGGACCTA-3′.

### Western blotting analysis.

Cultured HLMVECs were lysed with RIPA buffer (Santa Cruz Biotechnology, catalog sc-24948) supplemented with PMSF, protease inhibitor, and phosphatase inhibitor cocktail (Santa Cruz Biotechnology, catalog sc-45044). Protein samples were subjected to SDS-PAGE. After electrophoresis, separated proteins were transferred onto nitrocellulose membranes. The membranes were blocked in 5% nonfat milk for 1 hour and subsequently incubated with diluted primary antibodies at 4°C overnight. After primary antibody incubation, membranes were rinsed with TBS containing 0.1% Tween 20 for 3 times and then incubated with peroxidase-conjugated secondary antibodies for 1 hour at RT. After 3 washes, the membrane was incubated with ECL for 5 minutes, and the protein bands were detected by Molecular Imager ChemiDoc XRS + with Image Lab Software (Bio-Rad Laboratories). The intensities of the protein bands were quantified by ImageJ (NIH). The following primary antibodies were used in this study: anti-GCN2 (Abcam, catalog ab134053, 1:500), anti-GCN2 (Cell Signaling Technology, catalog 3302, 1:500), anti–phospho-Thr899-GCN2 (Invitrogen, catalog PA5-105886, 1:500), anti–phospho-Ser51-EIF2α (Abcam, catalog ab32157, 1:1,000), anti-EIF2α (Cell Signaling Technology, catalog 9722, 1:1,000), anti–endothelin-1 (Abcam, catalog ab117757, 1:1,000), and anti–HIF-2α (Novus Biologicals, Bio-Techne, catalog NB100-122, 1:500). Anti–β-actin (MilliporeSigma, catalog A2228, 1:10,000) or anti–α-tubulin (MilliporeSigma, catalog T5168, 1:10,000) was used as a loading control.

### ELISA analysis.

HLMVECs were seeded and transfected with siGCN2 or control siRNA in 6-well plates and cultured to reach approximately 70% confluence and then starved overnight in FBS-free endothelial cell basal medium. After starvation, cells were replaced with fresh serum-free endothelial cell basal medium and exposed to hypoxia or normoxia for 48 hours. Culture media were collected for assessment of secreted EDN1 protein levels with Endothelin-1 ELISA Kit (R&D Systems, Bio-Techne, catalog DET100) according to the manufacturer’s instructions.

### HLMVECs’ regulation of PASMC proliferation.

As described above in the *ELISA analysis* section, culture media collected from HLMVECs under normoxia or 48-hour hypoxia were added to serum-starved PASMCs under normoxia. BrdU was added at the same time for assessing cell proliferation. At 24 hours later, PASMCs were fixed for anti-BrdU immunostaining (BD Biosciences, catalog 347580) according to the manufacturer’s instructions.

### Histological assessment.

Mouse lung tissues were fixed in situ with 10% PBS-buffered formalin for 5 minutes with 20 cm H_2_O pressure followed by overnight in 10% formalin at RT and then processed for paraffin embedding and sectioning. Semithin lung sections (5 μm) were deparaffinized, dehydrated, and subjected to H&E staining or Russell-Movat pentachrome staining (American MasterTech, catalog KTRMP) according to the manufacturer’s instructions. Sections were imaged with ZEISS Axio microscope. For assessment of pulmonary arterial wall thickness, pulmonary arteries from 50 images at 20× original magnification were quantified by ImageJ. Wall thickness was calculated by the distance between internal wall and external wall divided by the distance between external wall and the center of lumen.

### Immunofluorescence staining.

For immunofluorescence staining of anti-Thr899 phospho-GCN2, formalin-fixed lung sections from IPAH patients and control donors, mice, and rats were deparaffinized and rehydrated according to standard protocols. Antigen retrieval was performed by boiling the slides in target retrieval buffer (Advanced Cell Diagnostics, Bio-Techne) for 15 minutes, followed by Proteinase Plus (Advanced Cell Diagnostics, Bio-Techne) treatment at 40°C for 30 minutes. After blocking with 0.1% Triton X-100 and 5% normal goat serum in PBS for 1 hour at RT, the sections were incubated with anti-Thr899 phospho-GCN2 (Invitrogen, catalog PA5-105886, 1:50) and anti-vWF (Invitrogen, catalog MA5-14029, 1:5) for human lung tissue, anti-CD31 (BD Biosciences, catalog 550274, 1:25) for mouse lung tissue, or anti-CD31 (Bio-Rad, catalog MCA1334G, 1:10) for rat lung tissue at 4°C overnight. After 3 rinsings with PBS, the sections were incubated with Alexa Fluor 594–conjugated and Alexa Fluor 488–conjugated secondary antibodies (Life Technologies) at RT for 1 hour. The slides were mounted with antifade mounting medium containing DAPI (Vector Laboratories, catalog H-1200).

Mouse lung tissues were perfused with PBS and embedded in OCT for cryosectioning. For immunofluorescence staining of proliferative SMCs and ECs, lung cryosections (5 μm) were fixed with 4% paraformaldehyde for 20 minutes at RT and then blocked with 0.1% Triton X-100 and 5% BSA in PBS at RT for 1 hour. After 3 rinsings with PBS, slides were incubated with anti-Ki67 antibody (Invitrogen, catalog MA5-14520, 1:50) and anti–α-SMA (Abcam, catalog ab8211, 1:300) or anti-CD31 (R&D Systems, Bio-Techne, catalog AF3628, 1:100) at 4°C overnight, then incubated with Alexa Fluor 594–conjugated anti-rabbit IgG (Life Technologies, catalog A-11012, 1:300) and Alexa Fluor 488–conjugated anti-goat IgG (Life Technologies, catalog A-11055, 1:300) at RT for 1 hour. The nuclei were counterstained with DAPI contained in antifade mounting medium. Images were taken with the ZEISS confocal microscope (LSM880) equipped with a plan-Apochromat 20×/0.8 M27 and a plan-Apochromat 63×/1.4 Oil DIC M27 objective lens.

To quantify muscularization of distal pulmonary vessels, lung cryosections were immunostained with anti–α-SMA (Abcam, catalog ab8211, 1:300) and mounted with antifade mounting medium with DAPI. Images were taken with the ZEISS confocal microscope (LSM880) equipped with a plan-Apochromat 20×/0.8 M27 objective lens. Twenty pictures of 20× fields were taken for each section (5–6 sections for each group). The total number of α-SMA–positive distal pulmonary vessels (d ≤ 50 μm) for each section was used for each mouse.

### Statistics.

Prism 8 (GraphPad Software, Inc.) was used for statistical analysis. Two-group comparisons were analyzed by the unpaired 2-tailed *t* test for normal distribution or Mann-Whitney *U* test for non-normal distribution. Multiple-group comparisons were analyzed by 1-way ANOVA followed by Holm-Šídák, Tukey’s, or Dunnett’s multiple comparisons test or 2-way ANOVA followed by Tukey’s multiple comparisons test. *P* value less than 0.05 denoted the presence of a statistically significant difference. All dot plot figures represent means + SD.

### Study approval.

All animals were handled according to the National Institutes of Health (NIH) *Guide for the Care and Use of Laboratory Animals* (National Academies Press, 2011), and the Northwestern University Institutional Animal Care and Use Committee approved protocols. Archived human lung tissue samples from patients with IPAH and control individuals were obtained from the Pulmonary Hypertension Breakthrough Initiative. The acquirement and handling of these samples were approved by the Institutional Review Board of Ann & Robert H. Lurie Children’s Hospital of Chicago.

### Data availability.

RNA-sequencing data were deposited at NCBI SRA database (SRA accession PRJNA994888). The data points shown in graphs are listed in an associated Supporting Data Values spreadsheet.

## Author contributions

MMZ and YYZ conceived the experiments. MMZ, JD, ZD, and YP designed experiments, carried out experiments, and analyzed the data. YYZ analyzed and interpreted the data. MMZ drafted the manuscript. YYZ supervised the project, revised the manuscript, and is responsible for the concept. All authors edited the manuscript.

## Supplementary Material

Supplemental data

Unedited blot and gel images

Supporting data values

## Figures and Tables

**Table 2:**
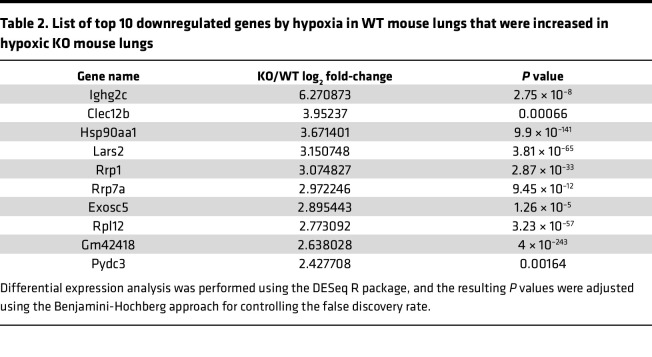
List of top 10 downregulated genes by hypoxia in WT mouse lungs that were increased in hypoxic KO mouse lungs

**Table 1:**
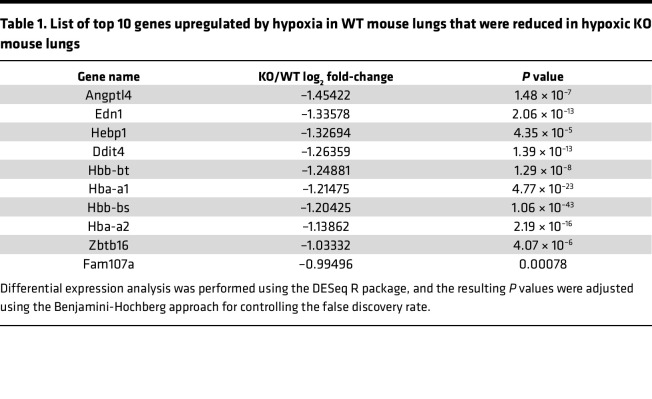
List of top 10 genes upregulated by hypoxia in WT mouse lungs that were reduced in hypoxic KO mouse lungs

## Version 1 09/24/2024

In-Press Preview

## Version 2 10/22/2024

Electronic publication

## References

[1] Simonneau G, Hoeper MM (2019). The revised definition of pulmonary hypertension: exploring the impact on patient management. Eur Heart J Suppl.

[2] Hoeper MM (2016). A global view of pulmonary hypertension. Lancet Respir Med.

[3] Humbert M (2004). Treatment of pulmonary arterial hypertension. N Engl J Med.

[4] McLaughlin VV (2009). ACCF/AHA 2009 expert consensus document on pulmonary hypertension: a report of the American College of Cardiology Foundation Task Force on Expert Consensus Documents and the American Heart Association: developed in collaboration with the American College of Chest Physicians, American Thoracic Society, Inc., and the Pulmonary Hypertension Association. Circulation.

[5] Galie N (2016). 2015 ESC/ERS guidelines for the diagnosis and treatment of pulmonary hypertension: the joint task force for the diagnosis and treatment of pulmonary hypertension of the European Society of Cardiology (ESC) and the European Respiratory Society (ERS): endorsed by: Association for European Paediatric and Congenital Cardiology (AEPC), International Society for Heart and Lung Transplantation (ISHLT). Eur Heart J.

[6] D’Alonzo GE (1991). Survival in patients with primary pulmonary hypertension. Results from a national prospective registry. Ann Intern Med.

[7] Galie N (2015). Initial use of ambrisentan plus tadalafil in pulmonary arterial hypertension. N Engl J Med.

[8] Benza RL (2012). An evaluation of long-term survival from time of diagnosis in pulmonary arterial hypertension from the REVEAL registry. Chest.

[9] Sitbon O (2016). Initial dual oral combination therapy in pulmonary arterial hypertension. Eur Respir J.

[10] Van de Veerdonk MC (2011). Progressive right ventricular dysfunction in patients with pulmonary arterial hypertension responding to therapy. J Am Coll Cardiol.

[11] Lau EMT (2017). Epidemiology and treatment of pulmonary arterial hypertension. Nat Rev Cardiol.

[12] Berlanga JJ (1999). Characterization of a mammalian homolog of the GCN2 eukaryotic initiation factor 2alpha kinase. Eur J Biochem.

[13] Sood R (2000). A mammalian homologue of GCN2 protein kinase important for translational control by phosphorylation of eukaryotic initiation factor-2alpha. Genetics.

[14] Harding HP (2000). Regulated translation initiation controls stress-induced gene expression in mammalian cells. Mol Cell.

[15] Yang R (2000). Glucose limitation induces GCN4 translation by activation of Gcn2 protein kinase. Mol Cell Biol.

[16] Rolfes RJ, Hinnebusch AG (1993). Translation of the yeast transcriptional activator GCN4 is stimulated by purine limitation: implications for activation of the protein kinase GCN2. Mol Cell Biol.

[17] Dong J (2000). Uncharged tRNA activates GCN2 by displacing the protein kinase moiety from a bipartite tRNA-binding domain. Mol Cell.

[18] Ruff M (1991). Class II aminoacyl transfer RNA synthetases: crystal structure of yeast aspartyl-tRNA synthetase complexed with tRNA(Asp). Science.

[19] Wek SA (1995). The histidyl-tRNA synthetase-related sequence in the eIF-2 alpha protein kinase GCN2 interacts with tRNA and is required for activation in response to starvation for different amino acids. Mol Cell Biol.

[20] Qiu H (1998). Dimerization by translation initiation factor 2 kinase GCN2 is mediated by interactions in the C-terminal ribosome-binding region and the protein kinase domain. Mol Cell Biol.

[21] Qiu H (2001). The tRNA-binding moiety in GCN2 contains a dimerization domain that interacts with the kinase domain and is required for tRNA binding and kinase activation. EMBO J.

[22] Romano PR (1998). Autophosphorylation in the activation loop is required for full kinase activity in vivo of human and yeast eukaryotic initiation factor 2alpha kinases PKR and GCN2. Mol Cell Biol.

[23] Wek RC (1992). Truncated protein phosphatase GLC7 restores translational activation of GCN4 expression in yeast mutants defective for the eIF-2 alpha kinase GCN2. Mol Cell Biol.

[24] Padyana AK (2005). Structural basis for autoinhibition and mutational activation of eukaryotic initiation factor 2alpha protein kinase GCN2. J Biol Chem.

[25] Qiu H (2002). Mutations that bypass tRNA binding activate the intrinsically defective kinase domain in GCN2. Genes Dev.

[26] Deng J (2002). Activation of GCN2 in UV-irradiated cells inhibits translation. Curr Biol.

[27] Zhan K (2004). Differential activation of eIF2 kinases in response to cellular stresses in Schizosaccharomyces pombe. Genetics.

[28] Liu Y (2010). Regulation of G(1) arrest and apoptosis in hypoxia by PERK and GCN2-mediated eIF2alpha phosphorylation. Neoplasia.

[29] Schmidt S (2019). A MYC-GCN2-eIF2α negative feedback loop limits protein synthesis to prevent MYC-dependent apoptosis in colorectal cancer. Nat Cell Biol.

[30] Ravindran R (2016). The amino acid sensor GCN2 controls gut inflammation by inhibiting inflammasome activation. Nature.

[31] Eyries M (2014). EIF2AK4 mutations cause pulmonary veno-occlusive disease, a recessive form of pulmonary hypertension. Nat Genet.

[32] Montani D (2009). Pulmonary veno-occlusive disease. Eur Respir J.

[33] Hadinnapola C (2017). Phenotypic characterization of EIF2AK4 mutation carriers in a large cohort of patients diagnosed clinically with pulmonary arterial hypertension. Circulation.

[34] Eichstaedt CA (2016). EIF2AK4 mutation as “second hit” in hereditary pulmonary arterial hypertension. Respir Res.

[35] Zhang X (2022). Robust genome editing in adult vascular endothelium by nanoparticle delivery of CRISPR-Cas9 plasmid DNA. Cell Rep.

[36] Hamanaka RB (2005). PERK and GCN2 contribute to eIF2alpha phosphorylation and cell cycle arrest after activation of the unfolded protein response pathway. Mol Biol Cell.

[37] Kimpe M (2012). Pkh1 interacts with and phosphorylates components of the yeast Gcn2/eIF2α system. Biochem Biophys Res Commun.

[38] Kim JW (2006). HIF-1-mediated expression of pyruvate dehydrogenase kinase: a metabolic switch required for cellular adaptation to hypoxia. Cell Metab.

[39] Najafov A (2011). Characterization of GSK2334470, a novel and highly specific inhibitor of PDK1. Biochem J.

[40] Kourembanas S (1991). Hypoxia induces endothelin gene expression and secretion in cultured human endothelium. J Clin Invest.

[41] Prie S (1997). The orally active ET(A) receptor antagonist (+)-(S)-2-(4,6-dimethoxy-pyrimidin-2-yloxy)-3-methoxy-3,3-diphe nyl-propionic acid (LU 135252) prevents the development of pulmonary hypertension and endothelial metabolic dysfunction in monocrotaline-treated rats. J Pharmacol Exp Ther.

[42] Satwiko MG (2015). Targeted activation of endothelin-1 exacerbates hypoxia-induced pulmonary hypertension. Biochem Biophys Res Commun.

[43] Giaid A (1993). Expression of endothelin-1 in the lungs of patients with pulmonary hypertension. N Engl J Med.

[44] Yamashita K (2001). Molecular regulation of the endothelin-1 gene by hypoxia. Contributions of hypoxia-inducible factor-1, activator protein-1, GATA-2, AND p300/CBP. J Biol Chem.

[45] Minchenko A, Caro J (2000). Regulation of endothelin-1 gene expression in human microvascular endothelial cells by hypoxia and cobalt: role of hypoxia responsive element. Mol Cell Biochem.

[46] Brazeau JF, Rosse G (2014). Triazolo[4,5-d]pyrimidine derivatives as inhibitors of GCN2. ACS Med Chem Lett.

[47] Zhang P (2002). The GCN2 eIF2alpha kinase is required for adaptation to amino acid deprivation in mice. Mol Cell Biol.

[48] Emanuelli G (2020). The integrated stress response in pulmonary disease. Eur Respir Rev.

[49] Nossent EJ (2018). Pulmonary vascular remodeling patterns and expression of general control nonderepressible 2 (GCN2) in pulmonary veno-occlusive disease. J Heart Lung Transplant.

[50] Lai YJ (2020). Unique wreath-like smooth muscle proliferation of the pulmonary vasculature in pulmonary veno-occlusive disease versus pulmonary arterial hypertension. J Formos Med Assoc.

[51] Evans CE (2021). Endothelial cells in the pathogenesis of pulmonary arterial hypertension. Eur Respir J.

[52] Dai Z (2018). Endothelial and smooth muscle cell interaction via FoxM1 signaling mediates vascular remodeling and pulmonary hypertension. Am J Respir Crit Care Med.

[53] Dai Z (2016). Prolyl-4 hydroxylase 2 (PHD2) deficiency in endothelial cells and hematopoietic cells induces obliterative vascular remodeling and severe pulmonary arterial hypertension in mice and humans through hypoxia-inducible factor-2α. Circulation.

