# Supplementary material for: GCN2 kinase activation mediates pulmonary vascular remodeling and pulmonary arterial hypertension
Source: JCI Insight. 2024 Sep 24;9(20):e177926. doi: 10.1172/jci.insight.177926 (PMC11530134; doi:10.1172/jci.insight.177926)
Supplement: Supplemental data [file jciinsight-9-177926-s073.pdf]

SUPPLEMENTAL MATERIALS

GCN2 Kinase Activation Mediates Pulmonary Vascular Remodeling and Pulmonary Arterial Hypertension

Maggie M. Zhu, Jingbo Dai, Zhiyu Dai, Yi Peng, and You-Yang Zhao

Supplemental Figures and Figure Legends

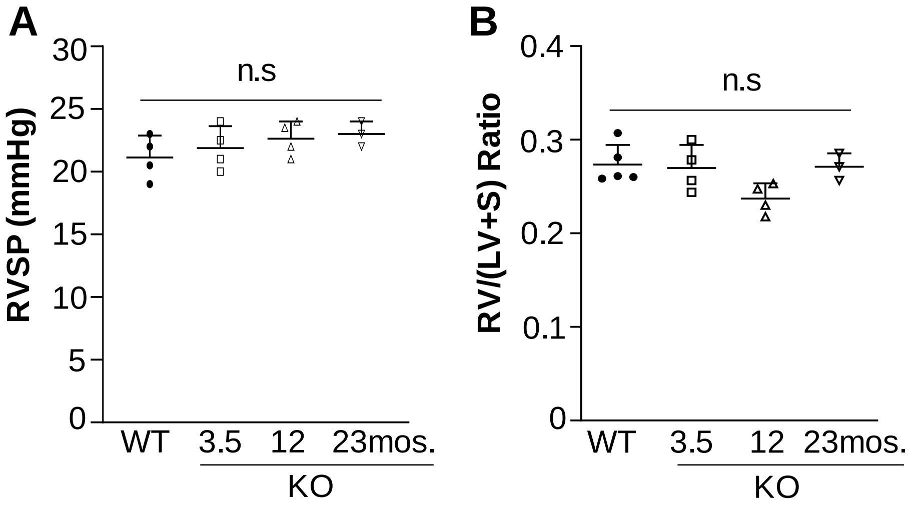

Supplemental Figure 1. GCN2 deficiency did not induce spontaneous PH in mice. (**A**) RVSP measurement showing no difference in 3.5 mos. old WT mice compared to KO mice at age of 3.5, 12, and 23 mos. (**B**) RV/LV+S ratio determination showing no difference between 3.5 mos. old WT mice and KO mice at various ages. N=3-5/group. Data are shown as means + SD. n.s=not significant. One-way ANOVA.

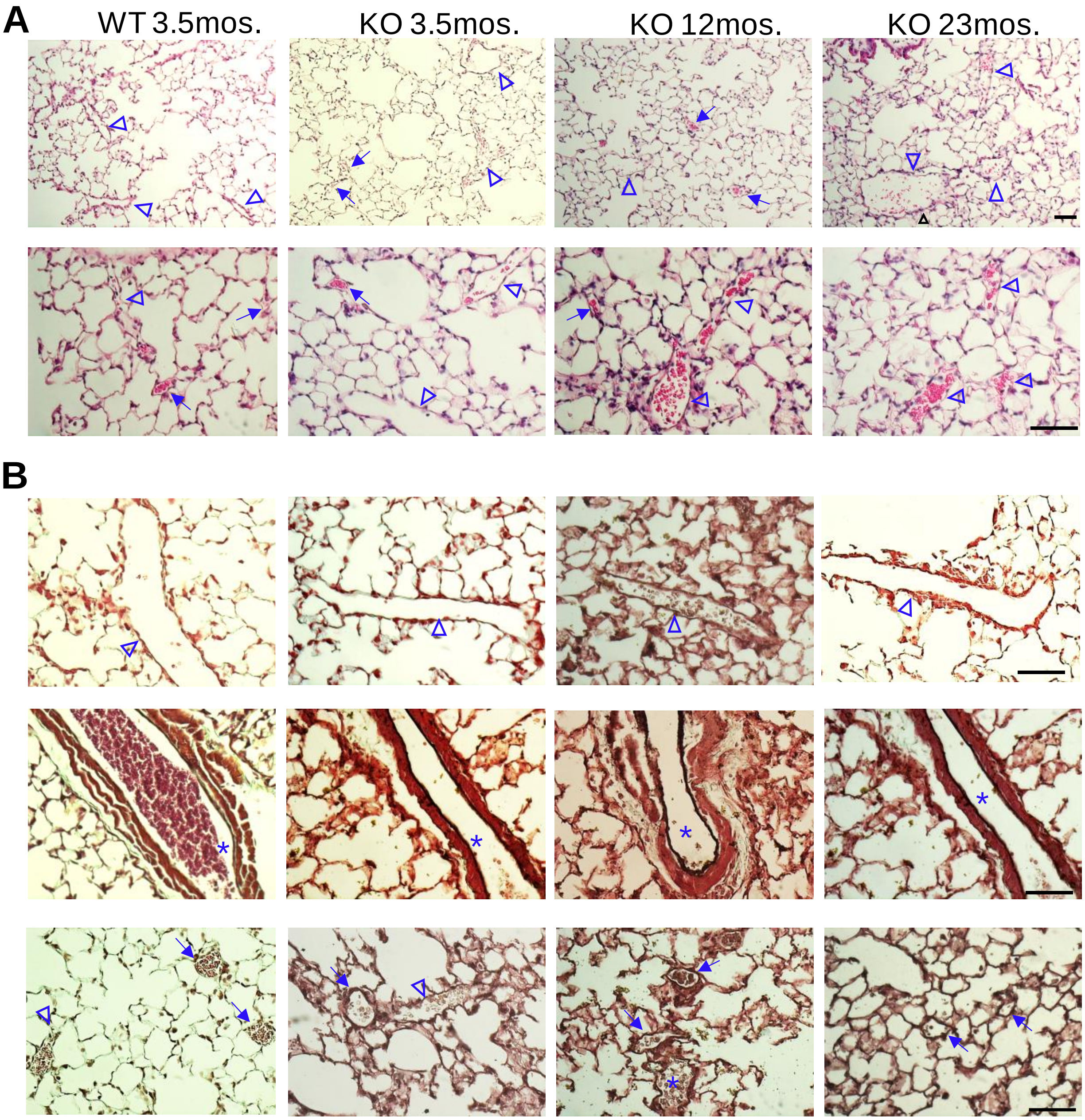

Supplemental Figure 2. Lack of characteristic pathological features of PVOD in lungs of KO mice. Representative micrographs of H&E staining (**A**) or Russel-Movat pentachrome staining (**B**) of lung sections. Normal structures of pulmonary arteries (asterisks) with thin muscular media, of pulmonary veins (triangles) with typical loose, slack walls and large lumens, and of microvessels including arterioles and venules (arrows) with thin vessel walls and clear lumens were exhibited in KO mice at various ages compared to WT mice (3.5 mos.). Scale bars, 50μm.

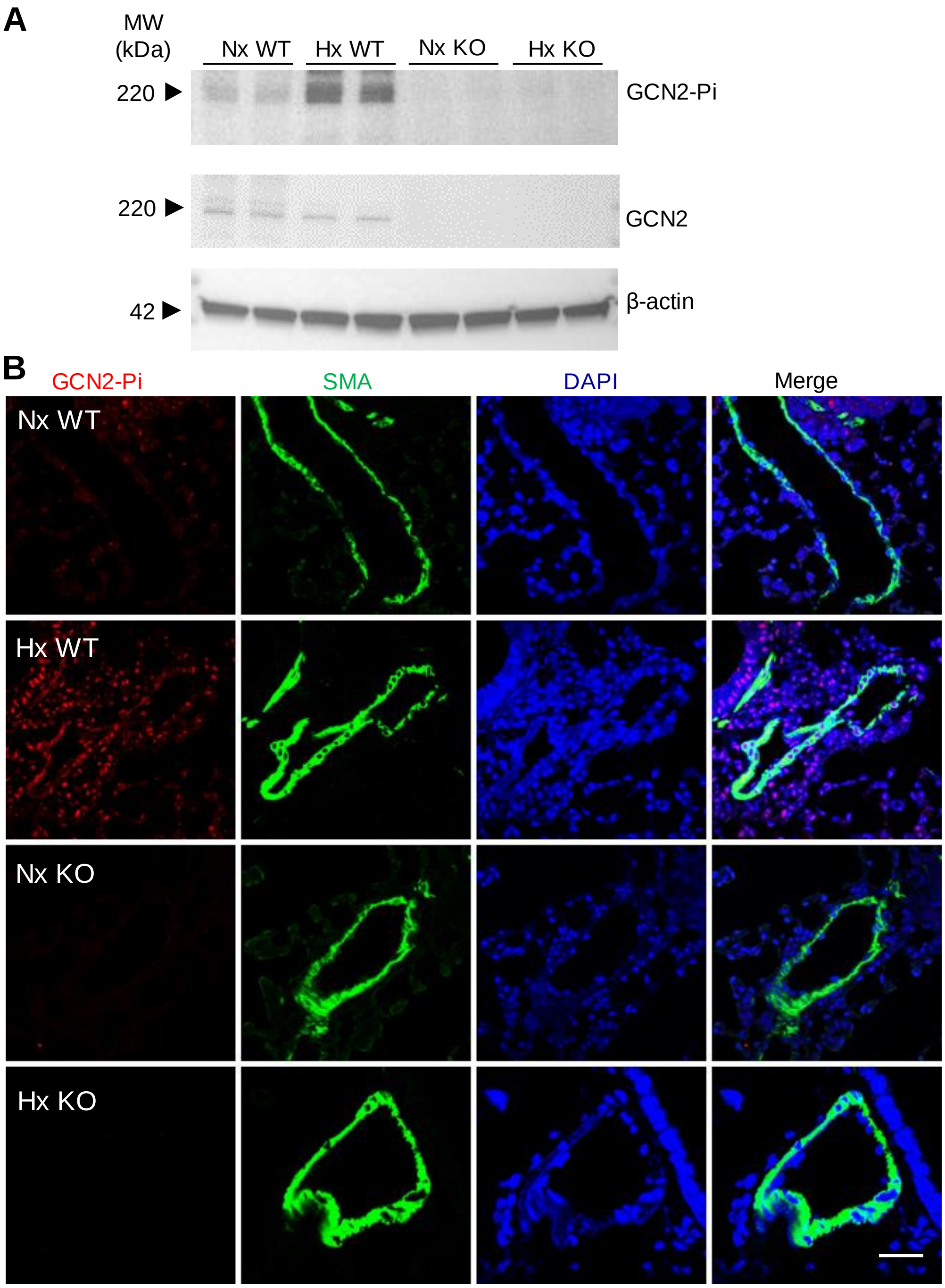

Supplemental Figure 3. (A) Western blotting showing GCN2 hyperphosphorylation in lungs of hypoxic WT (Hx WT) mice but not hypoxic KO mice (Hx KO). Nx=normoxia. (B) Representative micrographs of anti-phopho-GCN2 immunostaining of mouse lungs demonstrating prominent Thr898 phosphorylation-GCN2 (GCN2-Pi) in pulmonary arterial smooth muscle cells in hypoxic WT but not KO mice. Lung tissue cryosections from normoxic and hypoxic (3 weeks) WT mice and hypoxic KO mice were immunostained with anti-Thr phospho-GCN2 antibody (red). SMCs were immunostained with α-smooth muscle actin (SMA) (green) and nuclei were counterstained with DAPI (blue). Scale bar, 50μm.

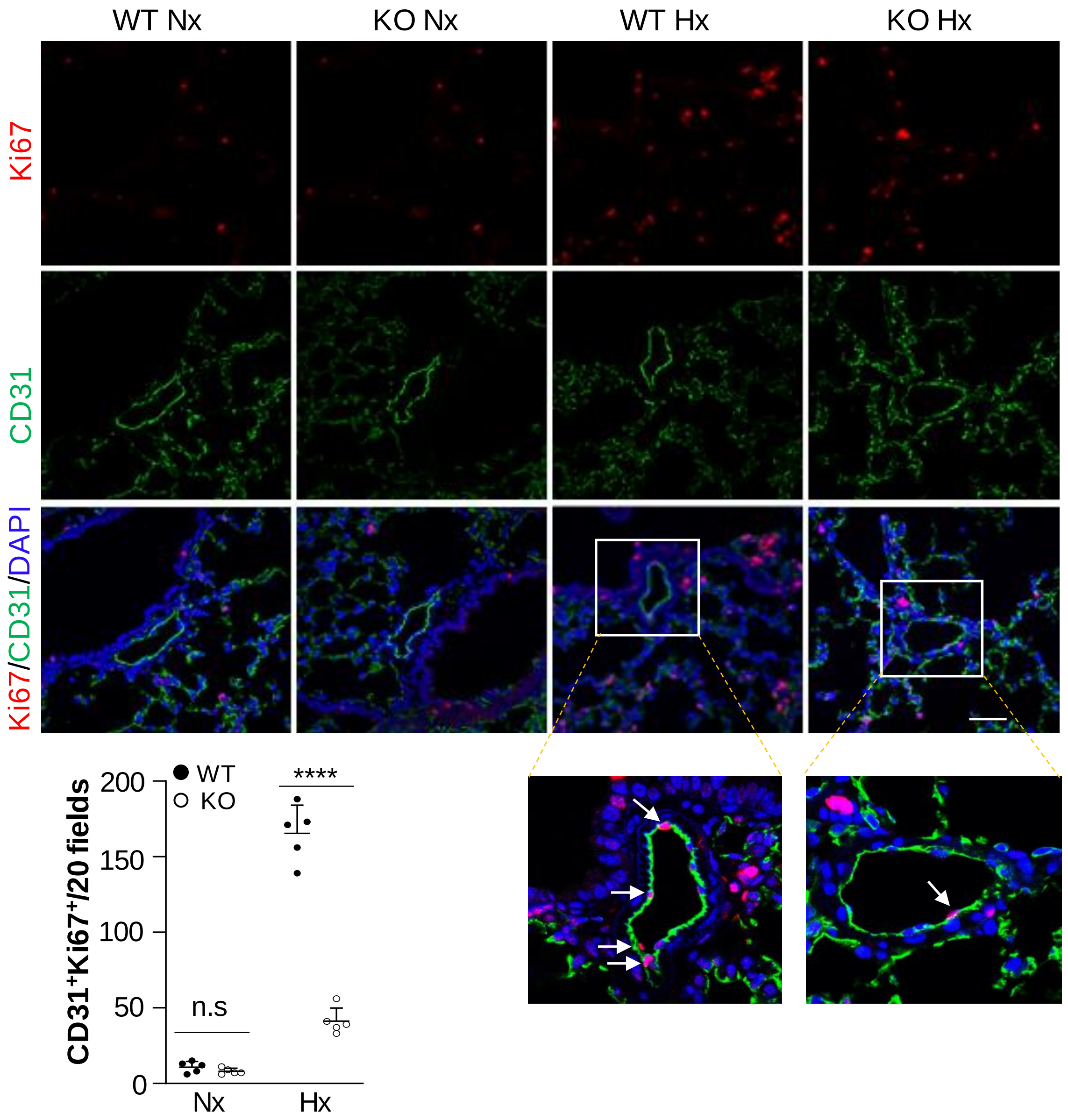

Supplemental Figure 4. Quantification of EC proliferation in mouse lungs. Mouse lungs were collected from 3.5 months old mice under normoxia (Nx) or 1 week hypoxia (Hx) for sectioning and immunostaining with anti-Ki67 (red) to identify proliferative cells. ECs were immunostained with anti-CD31 (green). Nuclei were counterstained with DAPI (blue). Scale bar, 50 µm. N=5/group. Data are shown as means + SD. n.s=not significant. ****, $P < 0.0001$. Two-way ANOVA with Tukey's multiple comparisons test.

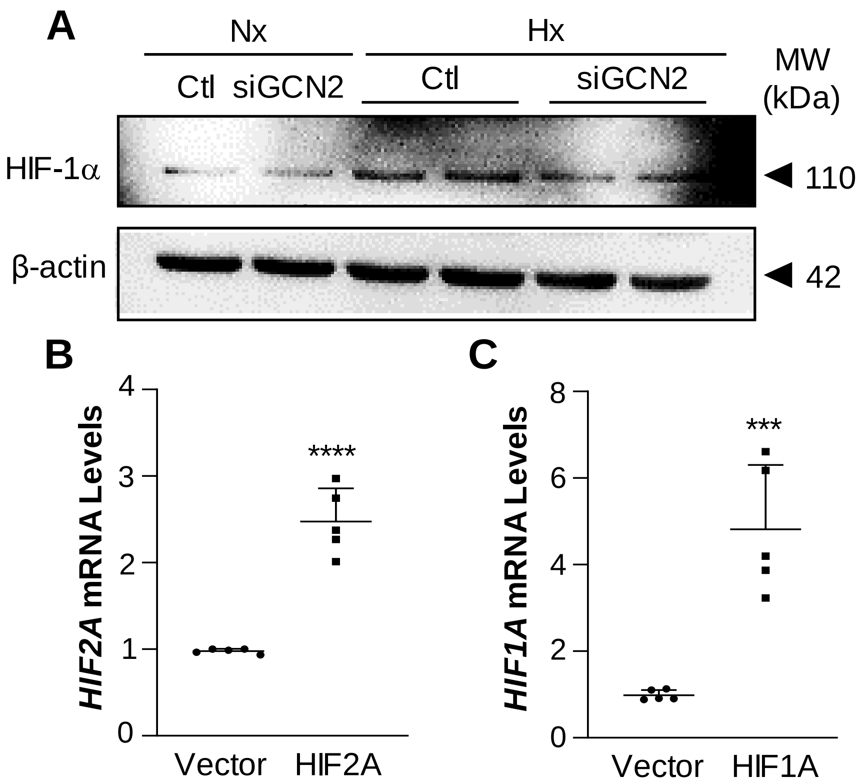

Supplemental Figure 5. Overexpression of HIFs by plasmid DNA transfection in cultured HLMVECs. (A) Western blotting demonstrating no significant difference in HIF-1α expression between hypoxic (Hx) GCN2-deficient HLMVECs and hypoxic control HLMVECs. Nx=normoxia. **(B, C)** Quantitative RT-PCR analysis demonstrating HIF plasmid DNA-transfection-mediated increases of *HIF1A* and *HIF2A* expression in HLMVECs. N=5/group. Data are shown as means + SD. ***, $P < 0.001$; ****, $P < 0.0001$. Unpaired two-tailed t test.

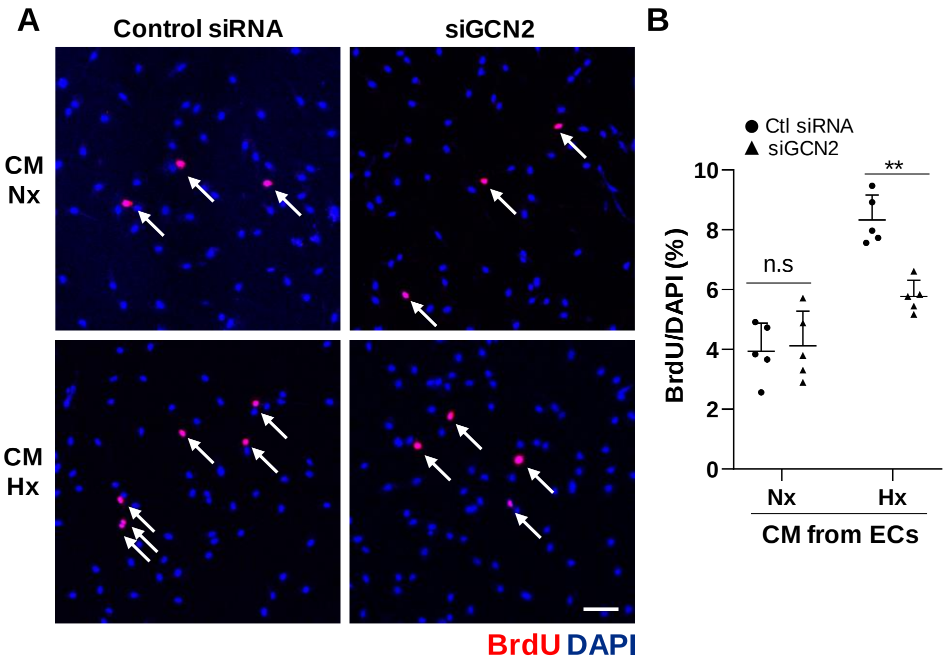

Supplemental Figure 6. Hypoxic EC-induced PASMC proliferation was GCN2-dependent. (**A**) Representative micrographs of BrdU immunostaining of PASMCs treated with EC conditioned medium (CM). Culture media from hypoxia (Hx) or normoxia (Nx) exposed GCN2-deficient (siGCN2) or control HLMVECs (Control siRNA) were collected and added to PASMC cultures under normoxia condition. BrdU was also added to PAMSC cultures at the same time. 24h later, PASMCs were fixed for immunostaining with anti-BrdU antibody (red). Nuclei were counterstained with DAPI (blue). Arrows point to proliferating PASMCs. Scale bar, 50µm. (**B**) Quantification of BrdU$^+$ PASMCs. N=5/group. Data are shown as means + SD. n.s=not significant. ** P < 0.01. Two-way ANOVA with Tukey's multiple comparisons test.

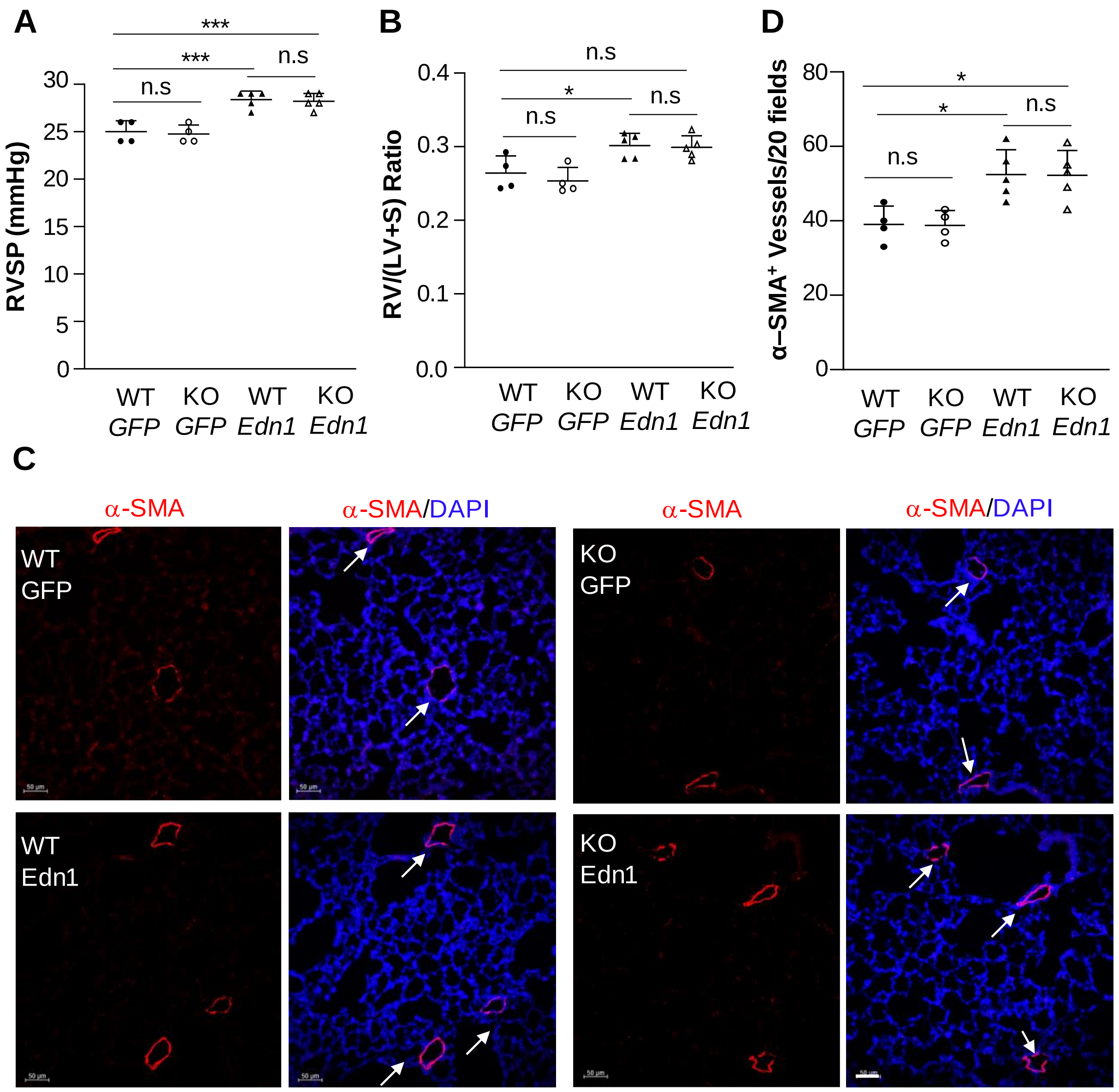

Supplemental Figure 7. Overexpression of Edn1 induced mild PH in both WT and KO mice under normoxia. (A, B) Increased RVSP and RV hypertrophy in both WT mice with *Edn1* plasmid and KO mice with *Edn1* plasmid compared to those with GFP plasmid. Edn1 plasmid or control GFP plasmid was delivered by EndoNP1 nanoparticles to WT and KO mice weekly for 3 weeks. **(C, D)** Representative micrographs of anti-α-SMA immunostaining of lung sections and quantification showing increases in the number of muscularized distal pulmonary vessels in both WT mice with *Edn1* plasmid and KO mice with *Edn1* plasmid compared to those with GFP plasmid. Arrows point to muscularized vessels. Scale bar, 50 µm. N=4-5/group. Data are shown as means + SD. n.s=not significant. n.s=not significant. *, $P < 0.05$; ***, $P < 0.001$. One-way ANOVA with Tukey's multiple comparisons test **(A, B, D)**.

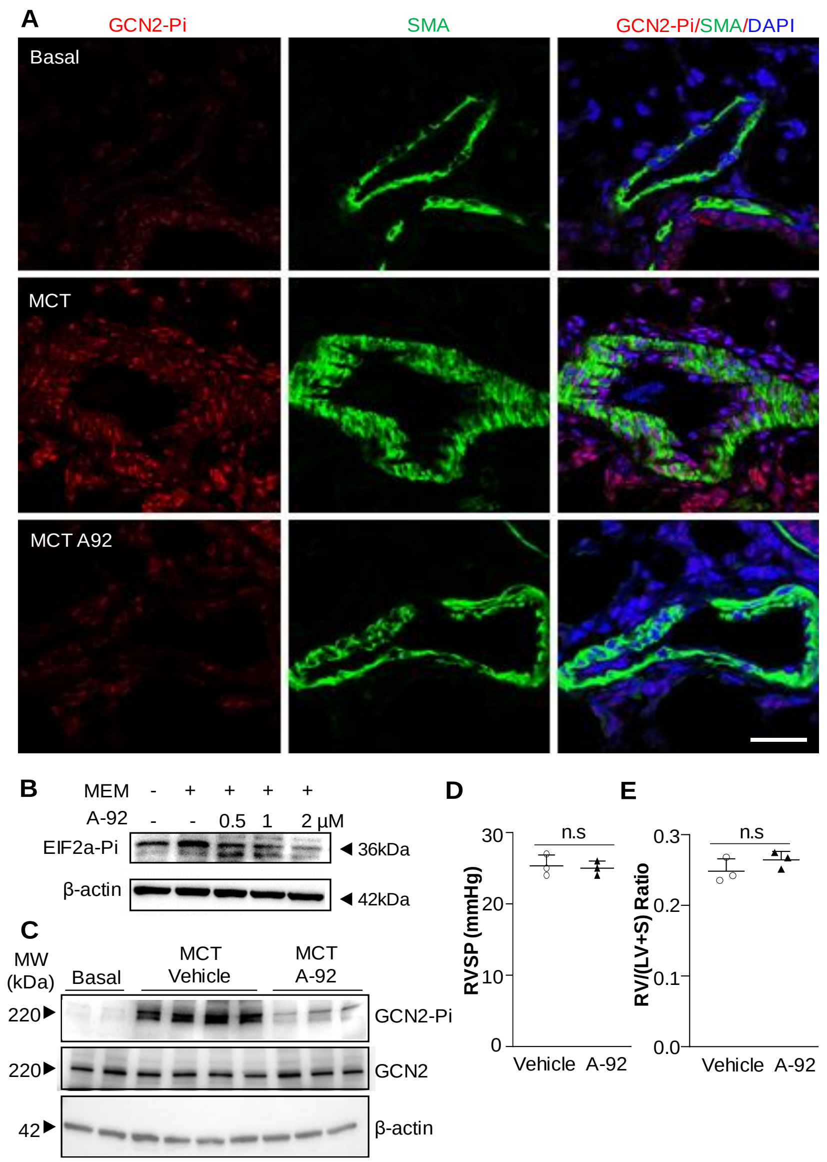

Supplemental Figure 8. GCN2 kinase inhibition by A-92 treatment in rats. (A) Representative micrographs of anti-GCN2-Pi immunostaining of rat lungs demonstrating prominent Thr898 phosphorylation-GCN2 (GCN2-Pi) in pulmonary arterial SMCs in vehicle-treated MCT rats, while largely inhibited in A-92 treated-MCT rats. Scale bar, 50µm. (**B**) Western blotting demonstrating A-92 inhibition of amino acid starvation-induced GCN2 phosphorylation in HLMVECs, confirming A-92 as a GCN2 kinase inhibitor. HLMVECs were exposed to minimal essential medium (MEM) lack of L-Glutamine to induce GCN2 activation and then treated with A-92. Total protein lysates were isolated for Western blotting with anti-Ser51 Phospho-EIF2a (EIF2a-Pi) antibody, while anti-β-actin was used as a loading control. (**C**) Western blotting demonstrating GCN2 phosphorylation was markedly increased in lung tissues of vehicle-treated MCT rats, whereas inhibited in A-92-treated MCT rats. **(D)** RVSP measurement showing no difference between vehicle- and A-92-treated rats without MCT challenge. (**E**) RV/LV+S ratio determination showing no effects of A-92 treatment on RV in rats without MCT challenge. N=3/group. Data are shown as means + SD. n.s=not significant.

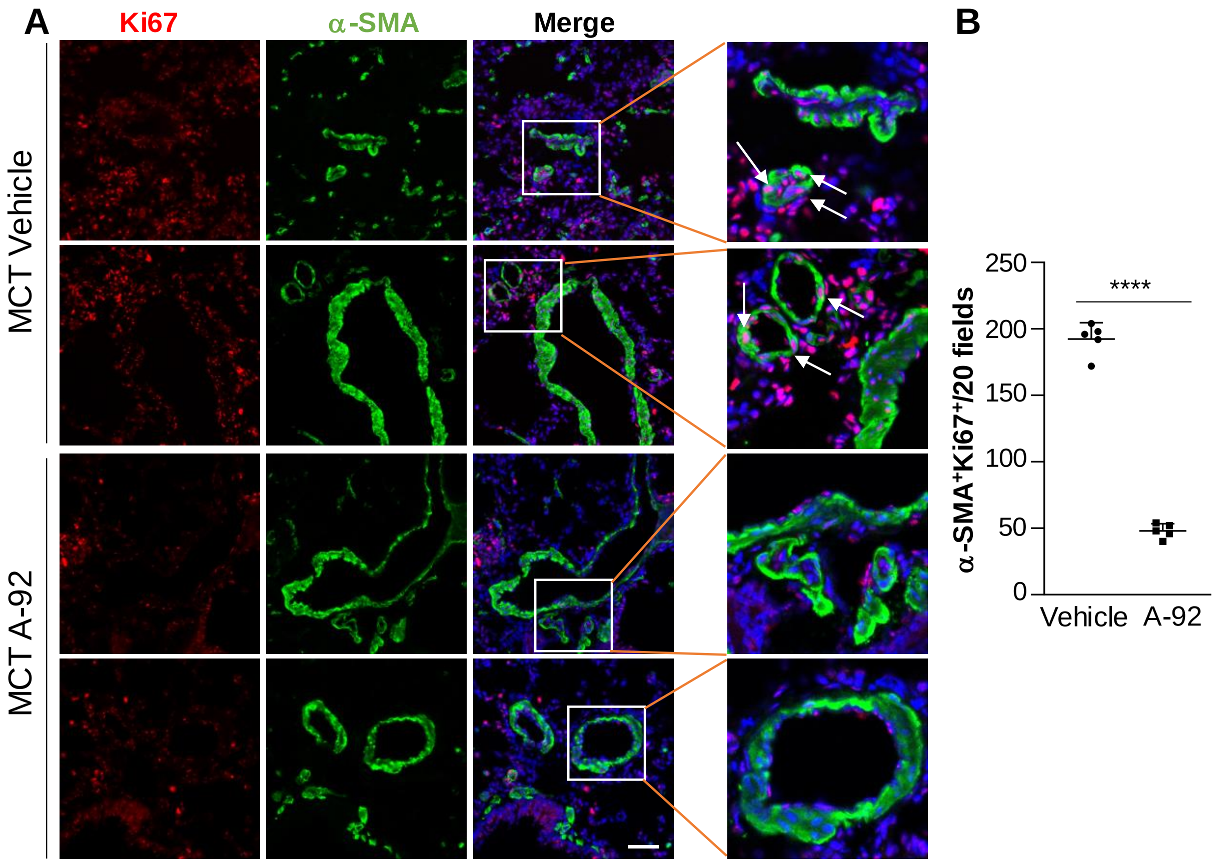

Supplemental Figure 9. Reduced pulmonary vascular SMC proliferation in A-92-treated MCT rats compared to vehicle-treated MCT rats. Rat lungs were collected from A-92 or vehicle-treated rats with MCT administration for cryosectioning and immunostaining with anti-Ki67 (red) to identify proliferative cells. SMCs were immunostained with anti-α-SMC. Nuclei were counterstained with DAPI (blue). (**A**) Representative micrographs of immunostaining of rat lung sections with anti-α-SMCs (green) and anti-Ki67. (**B**) Quantification of SMC proliferation. Arrows point to proliferating cells. Scale bar, 50 μm. N=5/group. Data are shown as means + SD. ****, $P < 0.0001$. Unpaired two-tailed t test.

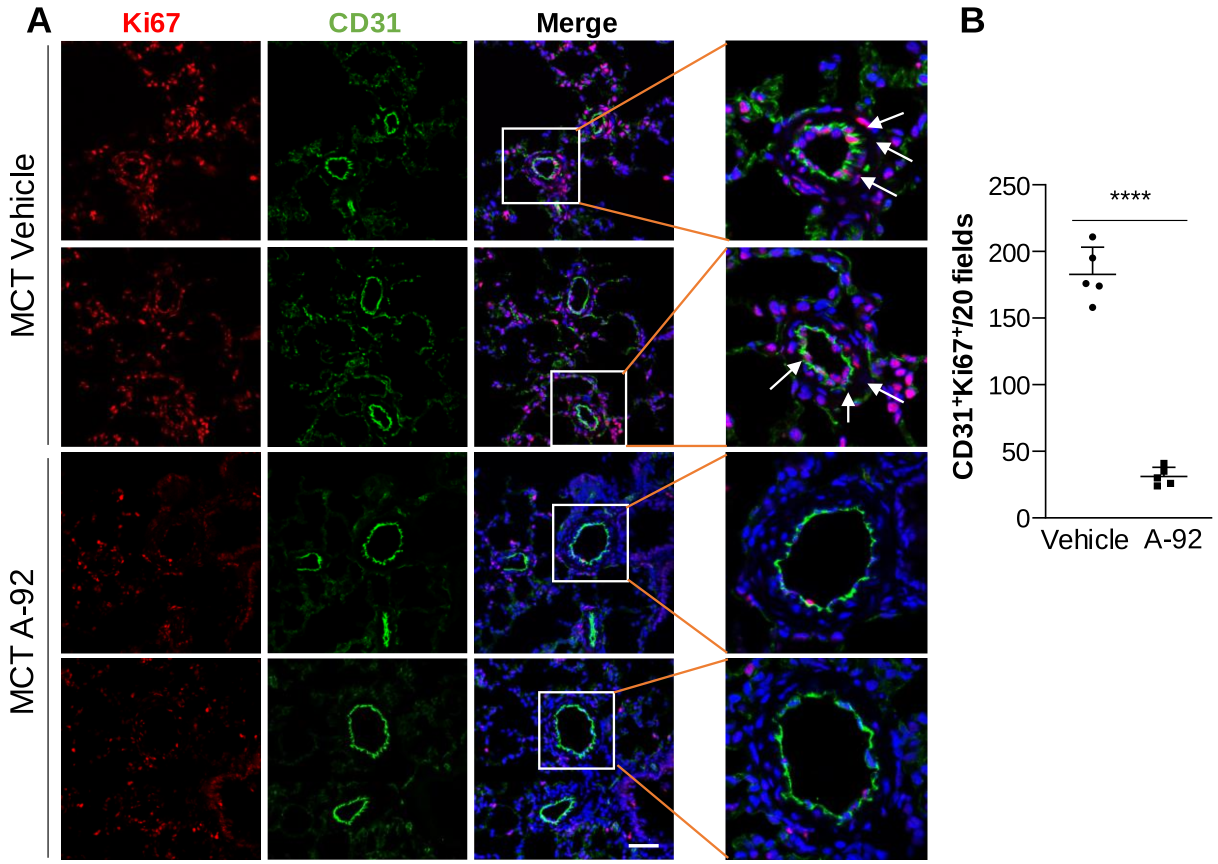

Supplemental Figure 10. Reduced pulmonary vascular EC proliferation in A-92-treated MCT rats compared to vehicle-treated MCT rats. Rat lungs were collected from A-92 or vehicle-treated rats with MCT administration for cryosectioning and immunostaining with anti-Ki67 (red) to identify proliferative cells. ECs were immunostained with anti-CD31 (green). Nuclei were counterstained with DAPI (blue). (**A**) Representative micrographs of immunostaining of rat lung sections with anti-CD31 (green) and anti-Ki67. (**B**) Quantification of EC proliferation. Arrows point to proliferating cells. Scale bar, 50 µm. N=5/group. Data are shown as means + SD. ****, P < 0.0001. Unpaired two-tailed t test.

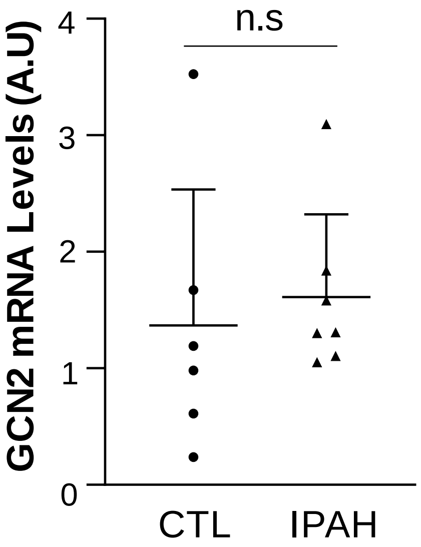

Supplemental Figure 11. Quantitative RT-PCR analysis demonstrating no difference of GCN2 mRNA expression between IPAH patient lungs and control normal lungs. Total RNA was isolated from lung tissues of IPAH patients and unused donors (CTL) for quantitative RT-PCR analysis. Control n=6, IPAH patients n=7. Data are shown as means + SD. A.U, arbitrary units. n.s=not significant. Mann-Whitney U test.

Supplemental Tables

Table 1. Demographics of IPAH patients and donors. Human lung tissue samples from IPAH patients and control subjects were obtained from the Pulmonary Hypertension Breakthrough Initiative (PHBI).

	**PHBI ID**	**Age**	**Race**	**Gender**
IPAH 1	CC 012	59YR	White	Female
IPAH 2	VA 011	32YR	White	Female
IPAH 3	BA 023	24YR	American Asian	Male
IPAH 4	UA 013	18YR	American Asian	Male
IPAH 5	ST 043	13YR	White	Male
IPAH 6	BA 017	40YR	White	Female
IPAH 7	ST 017	61YR	White	Male
	**PHBI ID**	**Age**	**Race**	**Gender**
Control 1	AH 025	54YR	Asian/Pacific Islander	Male
Control 2	AH 026	60YR	White	Female
Control 3	AH 019	52YR	White	Male
Control 4	AH 020	56YR	White	Female
Control 5	AH 027	45YR	White	Male
