# Supplementary material for: GCN2 kinase activation mediates pulmonary vascular remodeling and pulmonary arterial hypertension
Source: JCI Insight. 2024 Sep 24;9(20):e177926. doi: 10.1172/jci.insight.177926 (PMC11530134; doi:10.1172/jci.insight.177926)

Figure 3C

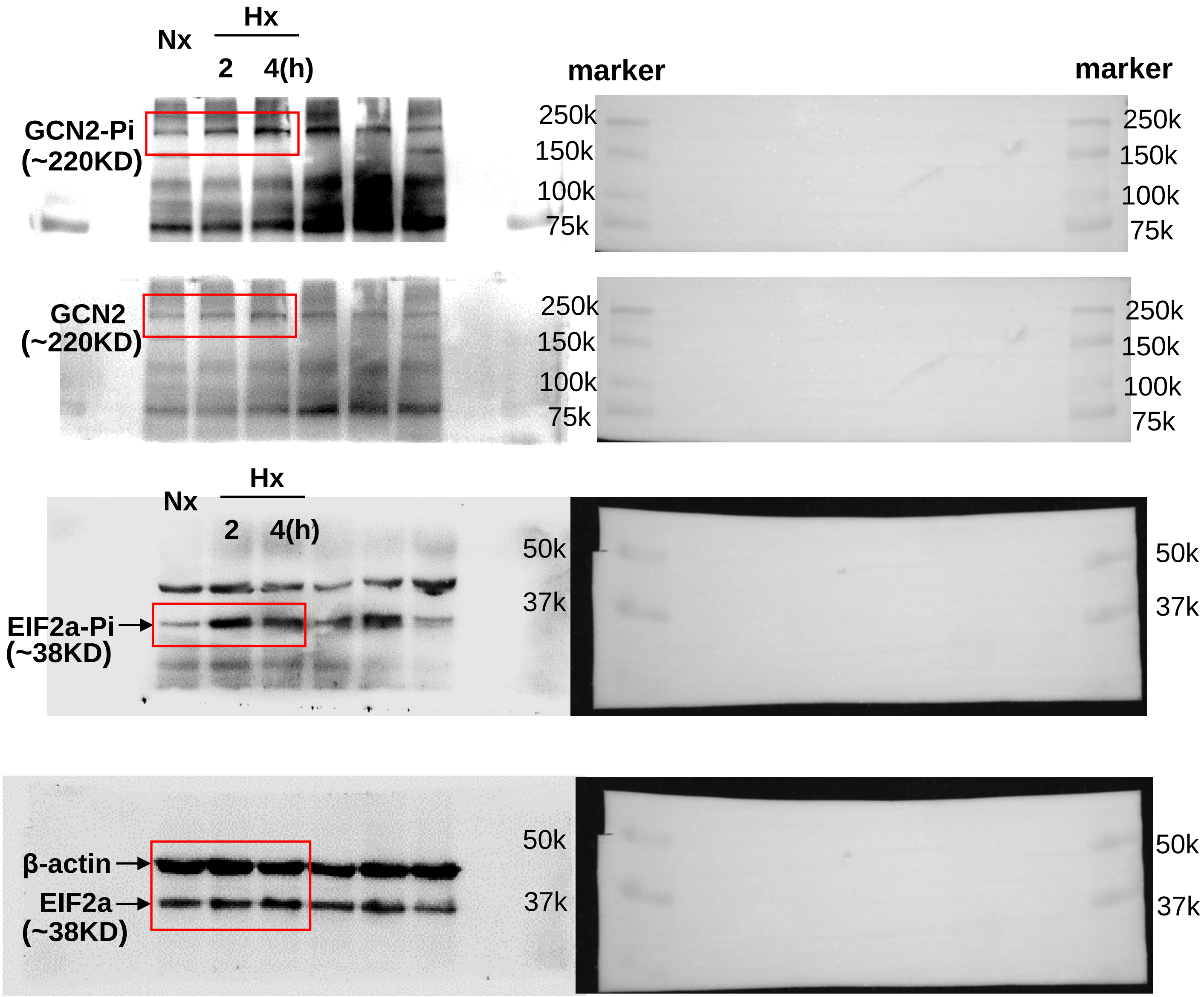

Figure 3H

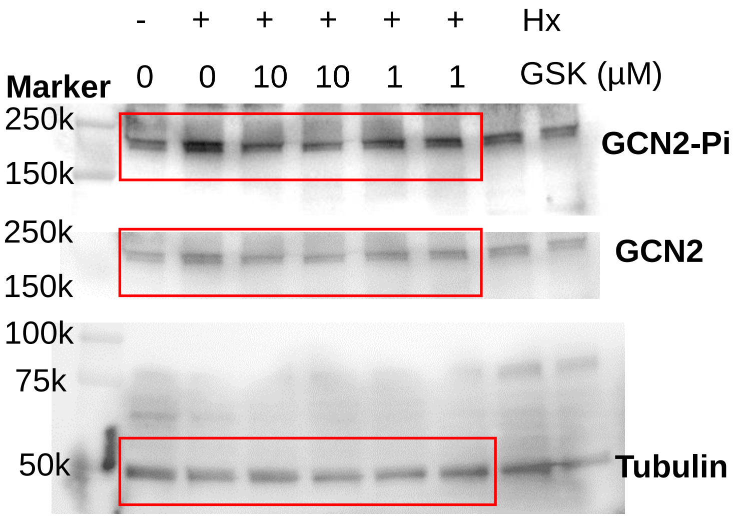

Figure 4B

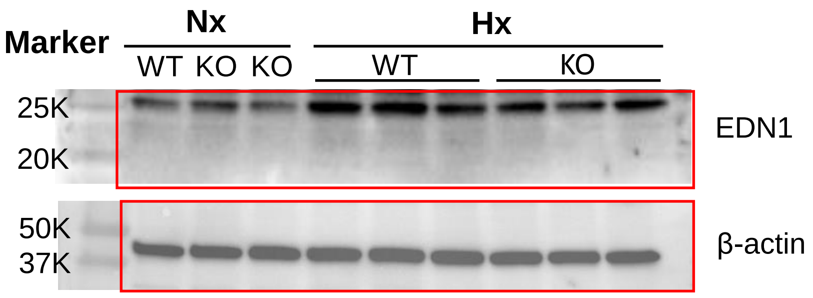

Figure 4G

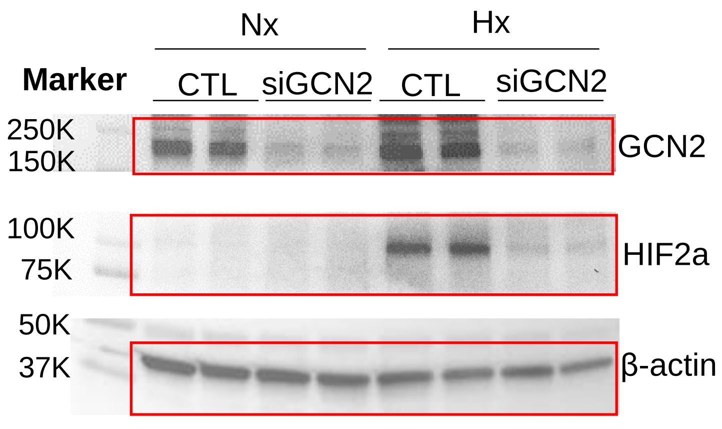

Figure 5B

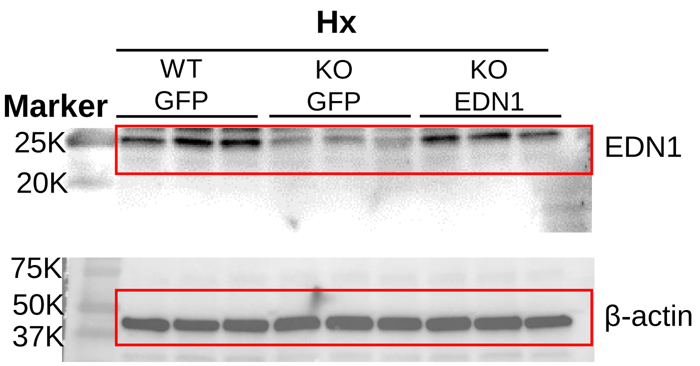

Figure 7A

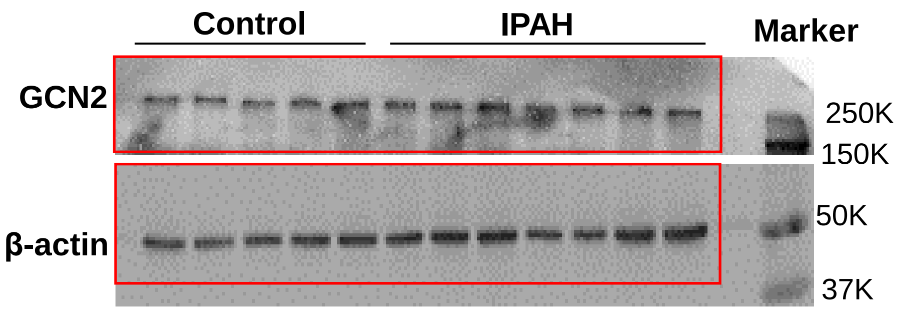

Figure 7C

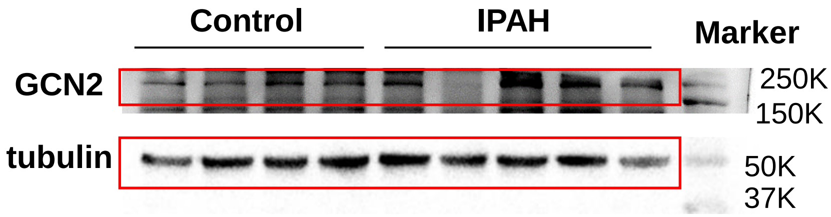

Figure 7E

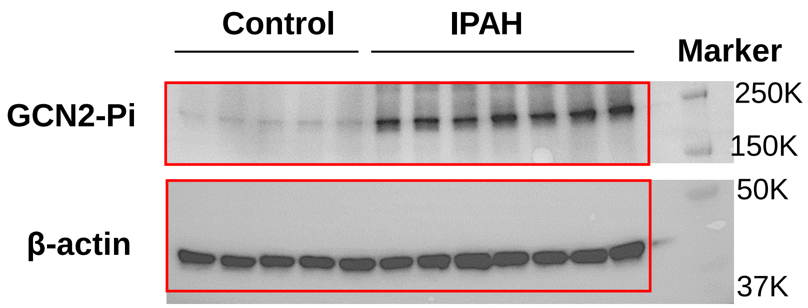

Supplemental **Figure 3A**

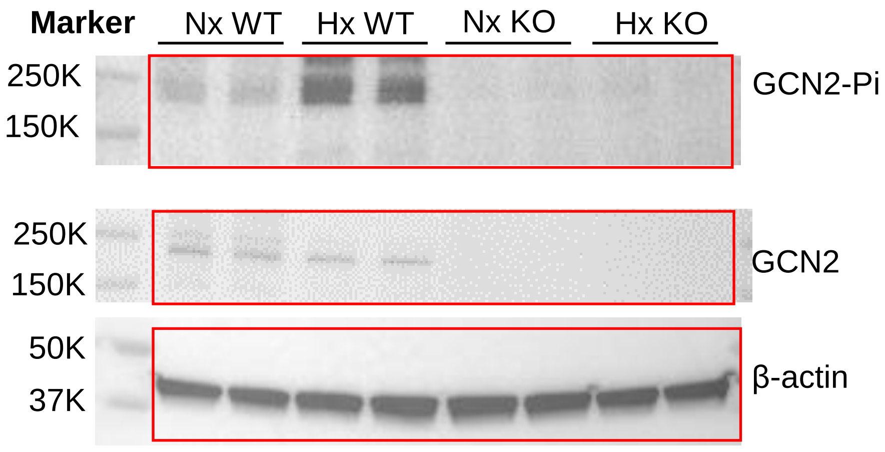

Supplemental **Figure 5A**

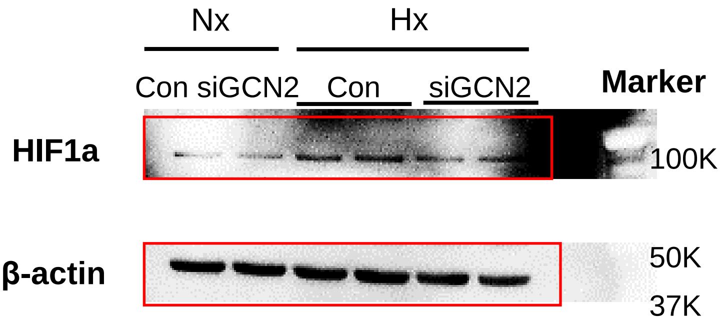

Supplemental Figure 8B

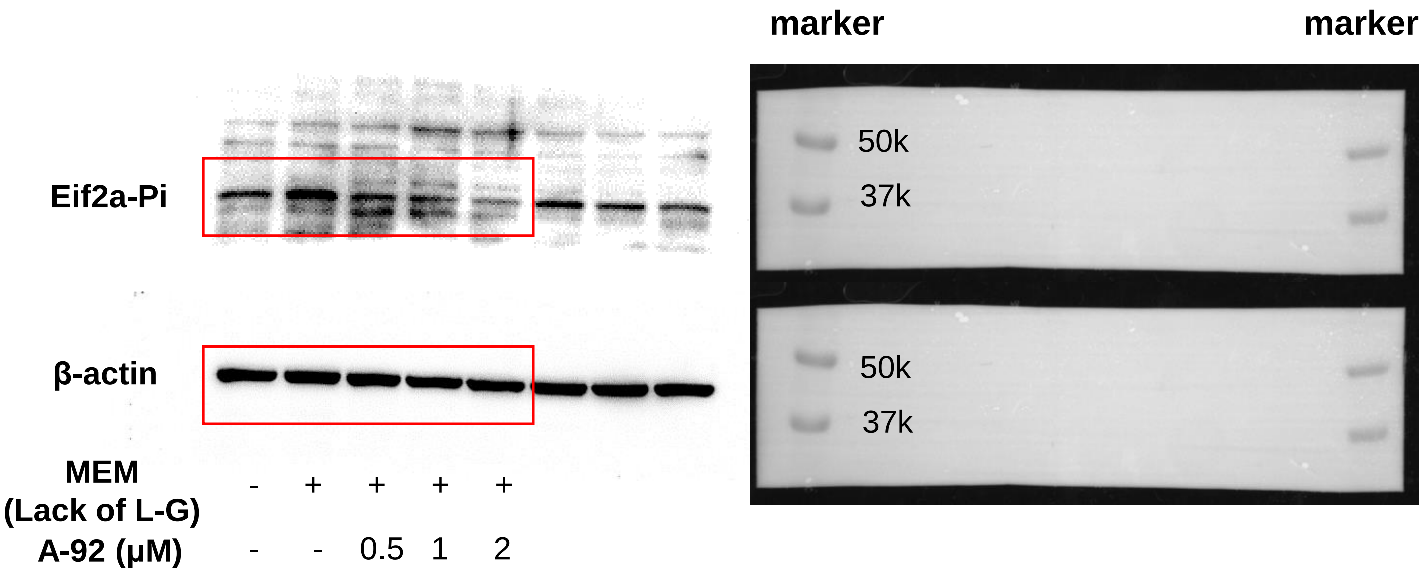

Supplemental Figure 8C

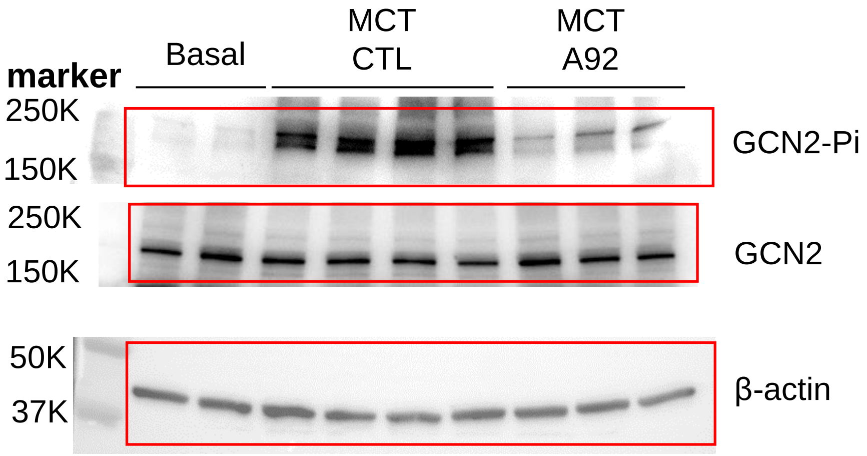

Supplement: Unedited blot and gel images [file jciinsight-9-177926-s074.pdf]
